# Supplementary material for: Analysis of News Media-Reported Snakebite Envenoming in Nepal during 2010–2022
Source: PLoS Negl Trop Dis. 2023 Aug 28;17(8):e0011572. doi: 10.1371/journal.pntd.0011572 (PMC10491300; doi:10.1371/journal.pntd.0011572)
Supplement: S1 Table — (DOCX) [file pntd.0011572.s001.docx]

| **S1 Table.** Names and languages used in the news media and URLs of articles included in this study. | | | |
| --- | --- | --- | --- |
| **SN** | **Name** | **Language** | **Numeric codes** given to the news articles included in this study are followed by URL accessed |
|  |  |  |  |
| 1 | Al Jazeera | English | **170709:** https://www.aljazeera.com/news/2017/7/9/nepali-teen-dies-from-snake-bite-in-menstruation-hut |
| 2 | Annapurna Post | Nepali | **150908:** http://annapurnapost.com/News.aspx/story/18747; **170803:** https://annapurnapost.com/news/76757-76757; **170903.a:** https://annapurnapost.com/news/79306-79306; **170903.b:** https://annapurnapost.com/news/79255-79255; **180702:** https://annapurnapost.com/news/mhottriimaa-srpko-ttokaaibaatt-ekaidin-caarjnaako-mrtyu-101949; **180711:** https://annapurnapost.com/news/kmlaakhonc-kssetrmaa-vissaalu-srpko-bigbigii-102695; **180715:** https://annapurnapost.com/news/gomn-srpko-ddsaaibaatt-mrtyu-103014; **180821:** https://annapurnapost.com/news/aaiisiiyuu-nhundaa-srpdnshko-upcaarmaa-smsyaa-106087; **180829:** https://annapurnapost.com/news/prdesh-1-srple-ddse-bhgvaan-bhrosaa-106786; **190709:** https://epaper.amn.media/annapurnapost-detail/750#gallery-4; **220817:** https://annapurnapost.com/news/agreement-3-210875 |
| 3 | BBC News | English & Nepali | **120823:** https://www.bbc.com/news/world-asia-19348834 (English); **220722:** https://www.bbc.com/nepali/news-62248693 (Nepali) |
| 4 | Bharosa Media | English | **200814:** https://bharosamedia.com/2020/08/14/woman-dies-from-snake-bite/ |
| 5 | businesspati.com | Nepali | **220628:** https://businesspati.com/सर्प र्पले ले-डसे सेर-कञ्च नपु पुरमा -ए/; **220811:** https://businesspati.com/नवलपरा सी मा -सर्प र्पले ले-डस्दा / |
| 6 | Chautari Post Online |  | **191218:** http://chautaripostonline.com/2019/12/8826/ |
| 7 | Chitwan Post | Nepali | **140611:** https://echitwanpost.com/12336/2014061102/50/46/; **160612:** Year: 19, Volume: 320, Number: 5384; **180908:** https://echitwanpost.com/73140/2018091022/44/44/ |
| 8 | chitwanaaja.com | Nepali | **220805:** https://www.chitwanaaja.com/19414 |
| 9 | Chitwansamachar.com | Nepali | **190623:** https://www.chitwansamachar.com/archives/7993? fbclid=IwAR3OfZjIIgqBGa7qmqAxhlqLOKnL5h2x8nyazDWIGdpnq1MKiN0YIVObifc |
| 10 | clickmandu.com | Nepali | **180719:** https://clickmandu.com/2018/07/55207.html |
| 11 | Dainik Nepalgunj | Nepali | **121009:** https://dailynepalgunj.files.wordpress.com/2012/10/2069-06-23-tuesday.pdf; **170724:** https://dailynepalgunj.files.wordpress.com/2018/12/2074-04-09.pdf; **170812:** https://dailynepalgunj.files.wordpress.com/2018/12/2074-04-28.pdf |
| 12 | deshsanchar.com | Nepali | **191022:** https://deshsanchar.com/2019/10/22/264315/; **211016:** https://deshsanchar.com/2021/10/16/568210/ |
| 13 | Dhanghadi Khabar | Nepali | **200927:** https://dhangadhikhabar.com/news/20639 |
| 14 | dhansanchar.com | Nepali | **210808:** https://dhansanchar.com/2021/08/10972 |
| 15 | diyopost.com | Nepali | **190508:** https://www.diyopost.com/05/58849/ |
| 16 | enepalpatra.com | Nepali | **220709:** https://www.enepalpatra.com/details/10209.html |
| 17 | gadhipost.com | Nepali | **220731:** https://www.gadhipost.com/2022/1060 |
| 18 | ghatanarabichar.com | Nepali | **190708:** https://www.ghatanarabichar.com/156589 |
| 19 | Global Press | English | **120910:** https://globalpressjournal.com/asia/nepal/seclusion-during-menstruation-continues-in-nepal-despite-supreme-court-ruling/ |
| 20 | gorkhapatraonline.com | English and Nepali | **English: 140613:** http://trn.gorkhapatraonline.com/index.php/nation/2427-cases-of-snake-bite-increase-in-sindhuli.html; **Nepali: 200621:** https://beta.gorkhapatraonline.com/open/2020-06-21-16926; **200704:** https://beta.gorkhapatraonline.com/index.php/open/2020-07-04-17840; **200818:** https://beta.gorkhapatraonline.com/open/2020-08-18-20768; **220609:** https://gorkhapatraonline.com/news/15447; **210618:** https://beta.gorkhapatraonline.com/arts/2021-06-18-40022 |
| 21 | Hamro Koshi Awaj | Nepali | **200611:** https://hamrokoshiawaz.com/archives/39245 |
| 22 | hamrodoctornews.com | Nepali | **170808:** https://www.hamrodoctornews.com/detail/6359 |
| 23 | healthykhabar.com | Nepali | **211016:** https://healthykhabar.com/posts/1163; **220128:** https://healthykhabar.com/posts/1823; **220615:** https://healthykhabar.com/posts/2641 |
| 24 | hernepati.com | Nepali | **210710:** https://www.hernepati.com/detail/8167 |
| 25 | hetaudanews.com | Nepali | **200715:** https://www.hetaudanews.com/2020/07/17013/ |
| 26 | himalayapost.com | Nepali | **180617:** https://himalayapost.com/archives/49858 |
| 27 | Himalkhabar.com | Nepali | **190816:** https://www.himalkhabar.com/news/14043; **191116:** https://www.himalkhabar.com/news/16089 |
| 28 | himalsanchar.com | English | **200701:** https://himalsanchar.com/one-lifeless-because-of-toxic-snake-chunk-in-kanchanpur/ |
| 29 | imagekhabar.com | English | **160628:** https://www.imagekhabar.com/news/150292/; **220806:** https://www.imagekhabar.com/news/332166/ |
| 30 | indrenionline.com | Nepali | **170520:** https://www.indrenionline.com/archives/30232 |
| 31 | informnepal.com | English | **210609:** https://informnepal.com/two-killed-from-snakebite-in-terai/ |
| 32 | Insec Online | Nepali | **170809:** https://inseconline.org/np/news/अस्पतालमै-धामीझाँक्रीको/; **191024:** https://inseconline.org/np/news/ |
| 33 | jagaranpost.com | Nepali | **190702:** https://jagaranpost.com/3713/47/ |
| 34 | Kanchuli Khabar | Nepali | **180705:** http://kanchulikkhabar.com/ |
| 35 | kanikakhabar.com | Nepali | **201009:** https://kanikakhabar.com/12970/ |
| 36 | Kantipur Samachar | Nepali | **100622:** https://ekantipur.com/national/2010/06/22/313522.html; **100623:** https://ekantipur.com/national/2010/06/23/313591.html; **100627:** https://ekantipur.com/national/2010/06/26/313729.html; **100703:** https://ekantipur.com/national/2010/07/03/314051.html; **100705:** https://ekantipur.com/national/2010/07/05/314147.html; **120626:** https://ekantipur.com/news/2012/06/26/350150.html; **120628:** https://ekantipur.com/news/2012/06/28/350263.html; **130702:** https://ekantipur.com/printedition/2013/07/02/299577.html; **130715:** http://www.ekantipur.com/np/2070/3/31/full-story/372152.html; **130727:** https://ekantipur.com/news/2013/07/27/372879.html; **150822:** https://ekantipur.com/ampnews/2015-08-22/20150822160346.html; **160618:** https://ekantipur.com/ampnews/2016-06-18/20160618163538.html; **160828:** https://ekantipur.com/printedition/2016/08/28/20160828185323.html; **160912:** https://ekantipur.com/printedition/2016/09/12/20160912104941.html; **180908:** https://ekantipur.com/news/2018/09/08/153636920163045795.html; **200609:** https://ekantipur.com/pradesh-5/2020/06/09/15917009645015624.html; **210619:** https://ekantipur.com/pradesh-1/2021/06/19/162407425906493769.html; **210707:** https://ekantipur.com/pradesh-7/2021/07/07/162563285853354837.html; **210801:** https://ekantipur.com/pradesh-2/2021/08/01/162778777206614373.html; **210813:** https://ekantipur.com/feature/2021/08/13/162884161320822175.html; **220611:** https://ekantipur.com/pradesh-7/2022/06/11/165491856701654218.html; **220714:** https://ekantipur.com/pradesh-5/2022/07/14/165776691748342406.html; **220728:** https://ekantipur.com/pradesh-6/2022/07/28/16589880011242701.html; **220729:** https://ekantipur.com/pradesh-1/2022/07/29/1659095624446729.html; **220730:** https://ekantipur.com/pradesh-7/2022/06/28/165638115471854600.html; **220907:** https://ekantipur.com/pradesh-6/2022/09/07/166255216073549162.html; **221006:** https://ekantipur.com/pradesh-1/2022/10/06/166504334104684690.html?author=1 |
| 37 | khabar.jp | Nepali | **140703:** http://khabar.jp/2014/07/bharatpur-hospital-developing-for-snake-bitten-patients/; **140708:** http://khabar.jp/2014/07/snakes-bite-a-girl-to-death/; **140804:** http://khabar.jp/2014/08/poisonous-snake-bite-a-bolbam-person-to-death/; **140826:** http://khabar.jp/2014/08/dead-after-poisomous-snake-bite/ |
| 38 | Khabarhub | English | **200522:** https://english.khabarhub.com/2020/22/98337/; **200621:** https://english.khabarhub.com/2020/21/106275/; **200818:** https://english.khabarhub.com/2020/18/120907/; **190903:** https://english.khabarhub.com/2019/03/42132/; **200921:** https://english.khabarhub.com/2020/21/129095/; **220805:** https://khabarhub.com/2022/05/418555/; **220910:** https://khabarhub.com/2022/10/431591/ |
| 39 | khaptadonline.com | Nepali | **190708:** https://www.khaptadonline.com/node/530 |
| 40 | Kharibot | Nepali | **190730:** https://www.kharibot.com/news-details/37781/Kharibot2015; **190903:** https://kharibot.com/news-details/39631/kharibot; **220907:** https://www.kharibot.com/news-details/119958/2022-09-07 |
| 41 | khojraftar.com | Nepali | **210826:** https://khojraftar.com/2021/08/26/17416 |
| 42 | koshipatra.com | Nepali | **220708:** https://koshipatra.com/news/2022/07/08/4792.html |
| 43 | kpkhabar.com | Nepali | **220803:** https://kpkhabar.com/2022/08/03/5359/ |
| 44 | ktmdainik.com | Nepali | **220621:** https://ktmdainik.com/2022/06/134307/ |
| 45 | lokaantar.com | Nepali | **210416:** https://lokaantar.com/story/140663/2021/4/16/current-affairs/snake# |
| 46 | lokpath.com | Nepali | **190706:** https://www.lokpath.com/story/99291 |
| 47 | madheshpradesh.com | Nepali | **190703:** https://madheshpradesh.com/mp-old/%E0%A4%A8%E0%A5%87%E0%A4%AA%E0%A4%BE%E0%A4%B2%E0%A5%80-%E0%A4%B8%E0%A5%87%E0%A4%A8%E0%A4%BE%E0%A4%95%E0%A5%8B-%E0%A4%B8%E0%A4%B9%E0%A4%AF%E0%A5%8B%E0%A4%97%E0%A4%AE%E0%A4%BE-%E0%A4%B8%E0%A4%AA/ |
| 48 | maipokharinews.com | Nepali | **170714:** maipokharinews.com/2017/07/jhapa-snake.html |
| 49 | merolifestyle.com | Nepali | **210614:** https://www.merolifestyle.com/archives/8165 |
| 50 | mirmireonline.com | Nepali | **150812:** https://www.mirmireonline.com/2015/08/12/11242/ |
| 51 | myRepublica | English | **150607:** http://www.myrepublica.com/society/story/22303/snakebite-cases-on-rise-in-tarai.html; **150721:** http://www.myrepublica.com/society/story/24938/two-die-in-separate-incidents-in-saptari.html; **150727:** http://www.myrepublica.com/society/story/25289/new-treatment-technique-helps-reduce-snakebite-deaths.html; **160517:** http://www.myrepublica.com/society/story/42487/man-strangulates-wife-to-death.html; **160811:** https://myrepublica.nagariknetwork.com/news/snakebites-kill-two-in-udayapur/; **160815:** https://myrepublica.nagariknetwork.com/news/snakebite-cases-on-the-rise/; **170713:** https://myrepublica.nagariknetwork.com/news/snakebite-cases-up-in-flood-hit-area/; **170801:** https://myrepublica.nagariknetwork.com/news/snakebite-deaths-rate-increasing-as-victims-arrive-late-for-treatment/; **170829:** https://myrepublica.nagariknetwork.com/news/snakebite-cases-rise-after-tarai-floods/; **180531:** https://myrepublica.nagariknetwork.com/news/snake-bite-claims-a-teenage-girl/; **180607:** https://myrepublica.nagariknetwork.com/news/snake-bite-claims-ward-chairman/; **180611:** https://myrepublica.nagariknetwork.com/news/woman-dies-after-snakebite-in-menstruation-shed/; **180617:** myrepublica.nagariknetwork.com/news/snakebite-menace-in-bardibas/; **180721:** https://myrepublica.nagariknetwork.com/news/1-103-people-bitten-12-killed-by-snakes-in-a-year/; **180724:** https://myrepublica.nagariknetwork.com/news/snakebite-treatment-centre-treats-1-407-cases-in-a-year/; **180826:** https://myrepublica.nagariknetwork.com/news/snakebite-cases-on-the-rise-in-itahari/; **190226:** https://myrepublica.nagariknetwork.com/news/over-500-patients-receives-treatment-from-snakebite-treatment-centre-arjuni/; **190526:** https://myrepublica.nagariknetwork.com/news/elderly-dies-of-snakebite-in-udayapur/; **190712:** https://myrepublica.nagariknetwork.com/news/snakebite-patients-in-trouble-as-treatment-center-remains-shut-for-three-days/; **190727:** https://myrepublica.nagariknetwork.com/news/snakebite-patients-in-eastern-nepal-dying-due-to-lack-of-timely-treatment/; **190814:** https://myrepublica.nagariknetwork.com/news/snakebite-patients-up-in-west-nawalparasi/; **220731:** https://myrepublica.nagariknetwork.com/news/32-year-old-youth-dies-after-not-receiving-treatment-for-snakebite/ |
| 52 | Nagarik News | Nepali | **170831:** https://nagariknews.nagariknetwork.com/social-affairs/128393-1504159500.html; **170901:** https://nagariknews.nagariknetwork.com/social-affairs/128445-1504245300.html; **171010:** https://nagariknews.nagariknetwork.com/social-affairs/130588-1507629000.html; **180702:** https://nagariknews.nagariknetwork.com/social-affairs/153179-1530510360.html; **180704:** https://nagariknews.nagariknetwork.com/social-affairs/153454-1530767880.html; **180707:** https://nagariknews.nagariknetwork.com/social-affairs/153621-1530974520.html; **180708:** https://nagariknews.nagariknetwork.com/social-affairs/153655-1531023300.html; **180711:** https://nagariknews.nagariknetwork.com/social-affairs/153920-1531285320.html; **180801:** https://nagariknews.nagariknetwork.com/social-affairs/155820-1533127620.html; **180820:** https://nagariknews.nagariknetwork.com/social-affairs/157505-1534772640.html; **180913:** https://nagariknews.nagariknetwork.com/social-affairs/159389-1536805980.html; **180920:** https://nagariknews.nagariknetwork.com/social-affairs/160017-1537435320.html; **200716:** https://nagariknews.nagariknetwork.com/social-affairs/285301-1595731556.html; **211001:** https://nagariknews.nagariknetwork.com/social-affairs/637331-1633084155.html; **220528:** https://nagariknews.nagariknetwork.com/social-affairs/837291-1653721376.html; **220821:** https://nagariknews.nagariknetwork.com/social-affairs/918561-1661081581.html |
| 53 | najiknews.com | Nepali | **210913:** https://najiknews.com/news/9279; |
| 54 | nayayougbodh.com | Nepali | **200524:** https://nayayougbodh.com/news/2020/05/24/348419 |
| 55 | Nepal Khabar | Nepali | **191011:** https://nepalkhabar.com/society/9687-2019-10-11-4-11-39; **220707:** https://nepalkhabar.com/madhesh/136408-2022-7-7-12-47-49 |
| 56 | Nepal24Hours | English | **190715:** https://nepal24hours.com/snakebite-cases-up-in-flood-hit-areas-across-nepal/snakebite-cases-up-in-flood-hit-areas-across-nepal-2/ |
| 57 | nepaliheadlines.com | Nepali and English | **220528: English:** https://nepaliheadlines.com/daughter-dies-of-snakebite-father-critical/; **Nepali:** **220708:** https://nepaliheadlines.com/झारफुकमा-भर-पर्दा-प्रेमश/; **220818:** https://nepaliheadlines.com/%E0%A4%B8%E0%A4%B0%E0%A5%8D%E0%A4%AA%E0%A4%B2%E0%A5%87-%E0%A4%9F%E0%A5%8B%E0%A4%95%E0%A5%8D%E0%A4%A6%E0%A4%BE-%E0%A4%8F%E0%A4%95%E0%A4%9C%E0%A4%A8%E0%A4%BE%E0%A4%95%E0%A5%8B-%E0%A4%9C%E0%A5%8D/ |
| 58 | nepalihealth.com | Nepali | **190717:** https://www.nepalihealth.com/2019/07/17/40376/ |
| 59 | nepallive.com | Nepali | **190816:** https://nepallive.com/story/128601; **220909:** https://nepallive.com/index.php/story/290698 |
| 60 | nepalnews.com | English | **211003:** https://nepalnews.com/s/nation/child-dies-of-snake-bite-in-mahottari; **210731:** https://nepalnews.com/s/issues/cases-of-snakebite-increased-in-jaleswar |
| 61 | nepalpage.com | Nepali | **220724:** https://www.nepalpage.com/2022/07/24/205863/ |
| 62 | nepalpostkhabar.com | Nepali | **170828**: https://nepalpostkhabar.com/2017/08/6788 |
| 63 | nepalpress.com | Nepali | **210716:** https://www.nepalpress.com/2021/07/26/81160/; **220821:** https://www.nepalpress.com/2022/08/21/225594/ |
| 64 | News24Nepal | Nepali | **170829:** https://www.news24nepal.tv/2017/08/29/174757; **180529:** https://www.news24nepal.tv/2018/05/29/334660; **190715:** https://www.news24nepal.tv/2019/07/15/544290 |
| 65 | newsmadhesh.com | Nepali | **190715:** https://www.newsmadhesh.com/details/1466.html |
| 66 | newssanjal.com | Nepali | **221006:** https://newssanjal.com/content/2022/10/06/24779/ |
| 67 | npkhabar24.com | Nepali | **190802:** https://www.npkhabar24.com/2019/100460/ |
| 68 | NPR news | English | **170710:** https://www.npr.org/sections/goatsandsoda/2017/07/10/536483652/banished-to-a-menstrual-shed-a-teen-in-nepal-is-bitten-by-a-snake-and-dies |
| 69 | Online Khabar | Nepali | **140825:** https://www.onlinekhabar.com/2014/08/182853; **140929:** https://www.onlinekhabar.com/2014/10/194993; **150602:** https://www.onlinekhabar.com/2015/06/284084; **150606:** https://www.onlinekhabar.com/2015/06/285390; **160816:** https://www.onlinekhabar.com/2016/08/466869; **170804:** https://www.onlinekhabar.com/2017/08/614894; **180612:** https://www.onlinekhabar.com/2018/06/685631; **200625:** https://www.onlinekhabar.com/2020/06/876406; **210718:** https://www.onlinekhabar.com/2021/07/985653; **220531:** https://www.onlinekhabar.com/2022/05/1135914; **220914:** https://www.onlinekhabar.com/2022/09/1190533 |
| 70 | Online Khabar | English | **190711:** https://english.onlinekhabar.com/shortage-of-anti-snake-venom-in-nuwakot.html; **210623:** https://english.onlinekhabar.com/snakebite-kills-1-in-bara.html; **210922:** https://english.onlinekhabar.com/saptari-snakebite.html; **220622:** https://english.onlinekhabar.com/teenage-girl-found-dead-in-rolpa.html |
| 71 | onlinepana.com | Nepali | **211016:** https://www.onlinepana.com/content/109166/ |
| 72 | pahilopost.com | Nepali | **200620:** https://pahilopost.com/content/20200620193237.html |
| 73 | paschimnepal.com | Nepali | **160901:** https://paschimnepal.com/2016/09/970/ |
| 74 | Press Pati | Nepali | **200919:** https://www.presspati.com/?p=9408 |
| 75 | purbelinews.com | Nepali | **221114:** https://purbelinews.com/533712 |
| 76 | purnapusti.com | Nepali | **210614:** https://purnapusti.com/%E0%A4%B8%E0%A4%B0%E0%A5%8D%E0%A4%AA%E0%A4%95%E0%A5%8B-%E0%A4%9F%E0%A5%8B%E0%A4%95%E0%A4%BE%E0%A4%88%E0%A4%AC%E0%A4%BE%E0%A4%9F-%E0%A4%8F%E0%A4%95%E0%A4%9C%E0%A4%A8%E0%A4%BE%E0%A4%95%E0%A5%8B/ |
| 77 | purwanchaldaily.com | Nepali | **190803:** https://purwanchaldaily.com/wp-content/uploads/2019/06/Sawan-18.pdf |
| 78 | radiosiddhababa.org.np | Nepali | **190712:** https://radiosiddhababa.org.np/news-details/1267/2019-07-12 |
| 79 | rajdhanidaily.com | Nepali | 200619: https://rajdhanidaily.com/id/15240/ |
| 80 | rajmargonline.com | Nepali | **220719:** https://rajmargonline.com/archives/40959 |
| 81 | reportersnepal.com | Nepali | **220722:** https://www.reportersnepal.com/2022/07/708054 |
| 82 | Ruru Khabar |  | **180717:** https://rurukhabar.wordpress.com/2018/07/17/ |
| 83 | Sadarline.com | Nepali | **180720:** https://sadarline.com/2018/07/19010 |
| 84 | safalkhabar.com | Nepali | **200610:** https://www.safalkhabar.com/news/45423; **200625:** https://www.safalkhabar.com/news/46325; **200722:** https://www.safalkhabar.com/news/47863; **200725:** https://www.safalkhabar.com/news/48041; **200808:** https://www.safalkhabar.com/news/48822; **200919:** https://www.safalkhabar.com/news/51259; **201014:** https://www.safalkhabar.com/news/52577; **210731:** https://www.safalkhabar.com/news/72309; **210801:** https://www.safalkhabar.com/news/72380; **220724:** https://www.safalkhabar.com/news/100323; **220906:** https://www.safalkhabar.com/news/104690; **221010:** https://www.safalkhabar.com/news/106789 |
| 85 | sajhasabal.com | Nepali | 160607: http://sajhasabal.com/nawalparasi/news/snake-bite-kumarworti/#sthash.h21LUnH1.dpuf |
| 86 | sancharkarmi.com | Nepali | **210908:** https://sancharkarmi.com/news-details/60874/2021-09-08 |
| 87 | Satyapati | Nepali | **180903:** https://satyapati.com/2018/09/15550/ |
| 88 | setokhabar.com | Nepali | **201015:** https://www.setokhabar.com/news/179638 |
| 89 | setopati.com | Nepali | **141030:** http://setopati.com/samaj/19296/; **150605:** https://www.setopati.com/samaj/29110/; **160822:** https://www.setopati.com/samaj/51913/; **180717:** https://www.setopati.com/social/163550; **180807:** https://www.setopati.com/index.php/social/164757 |
| 90 | setopati.net | Nepali | **140915:** http://m.setopati.net/news/3228/ |
| 91 | shilapatra.com | Nepali | **200629:** https://shilapatra.com/detail/33400; **220621:** https://shilapatra.com/detail/88982 |
| 92 | simanakhabar.com | Nepali | **220618:** https://www.simanakhabar.com/953 |
| 93 | sudurkhabar.com | Nepali | **190617:** https://sudurkhabar.com/archives/83881 |
| 94 | supadeuralikhabar.com | Nepali | **210618:** https://supadeuralikhabar.com/2021/06/18/10188/ |
| 95 | Swadesh Nepal | Nepali | **180913:** https://www.swadeshnepal.com/2018/09/31759/ |
| 96 | Swasthya Khabar Patrika | Nepali | **170829:** https://swasthyakhabar.com/; **210718:** https://staging.swasthyakhabar.com/story/41123 |
| 97 | Texas Nepal | English | **140716:** https://www.texasnepal.com/increasing-snake-bite-cases-in-kathmandu/ |
| 98 | Thaha Khabar | Nepali | **180623:** https://www.thahakhabar.com/news/42632/; **180716:** https://www.thahakhabar.com/news/44582/; **190617:** https://thahakhabar.com/election/contestant/72117/; **190708:** http://thaha4.prixa.net/news/73854/ |
| 99 | The Exclusive Weekly | Nepali | **130709**: http://theexclusiveweekly.blogspot.com/2013/07/blog-post_1689.html |
| 100 | The Himalayan Times | English | **150627:** https://thehimalayantimes.com/nepal/minor-dies-of-snakebite-in-sunsari; **150703:** http://thehimalayantimes.com/nepal/teen-dies-of-snakebite-in-jhapa/; **150707:** https://thehimalayantimes.com/nepal/snakebite-claims-one-in-kanchanpur; **150712:** http://thehimalayantimes.com/nepal/minor-dies-of-snake-bite-in-doti/; **150723.i:** https://thehimalayantimes.com/nepal/snakebite-kills-rautahat-girl/; **150723.ii:** https://thehimalayantimes.com/nepal/11-snake-bite-cases-in-a-day; **150726:** https://thehimalayantimes.com/nepal/number-of-snakebite-cases-up-in-jhapa/; **150728:** https://thehimalayantimes.com/nepal/snake-menace-in-camps-for-quake-survivors-in-bhaktapur-two-bitten/; **150820:** https://thehimalayantimes.com/nepal/snakebite-incidents-up-in-saptari/; **150826:** https://thehimalayantimes.com/nepal/man-dies-of-snake-bite-in-taplejung/; **150829:** https://thehimalayantimes.com/nepal/snakebite-death/; **160413:** https://thehimalayantimes.com/nepal/child-dies-snake-bite-rautahat; **160818:** https://thehimalayantimes.com/nepal/snakebite-claims-two-mahottari; **160825:** https://thehimalayantimes.com/nepal/snake-bite-cases-rising-temperature; **160906:** https://thehimalayantimes.com/kathmandu/man-dies-snake-bite; **170707:** https://thehimalayantimes.com/nepal/two-killed-snake-bite-rautahat; **170820:** https://thehimalayantimes.com/nepal/diseases-break-flood-hit-areas; **170822:** https://thehimalayantimes.com/nepal/cases-snake-bite-rise-saptari-district/; **171025:** https://thehimalayantimes.com/nepal/snake-rampage-scares-locals-in-kanchanpur; **180707**: https://thehimalayantimes.com/nepal/3-minor-girls-die-of-cobra-bites-in-a-week; **180730:** https://thehimalayantimes.com/nepal/snakebite-treatment-relieves-patients; **180810:** https://thehimalayantimes.com/nepal/antivenom-crunch-in-udayapur-health-facilities-hit-snakebite-patients; 180904: https://thehimalayantimes.com/nepal/snakebite-patients-up-in-tarai-region; **190716:** https://thehimalayantimes.com/nepal/snakebite-cases-up-in-flood-hit-areas; **190723:** https://thehimalayantimes.com/nepal/effective-service-in-place-for-snakebite-victims-in-jhapa-district; **190823:** https://thehimalayantimes.com/nepal/children-distraught-as-their-mother-dies-from-snakebite-in-kailali; **200723:** https://thehimalayantimes.com/nepal/udayapur-teenager-succumbs-to-snakebite-in-course-of-treatment; **200730:** https://thehimalayantimes.com/nepal/youth-dies-of-snake-bite-while-staying-in-quarantine; **200803:** https://thehimalayantimes.com/nepal/snakebite-patients-up-in-bheri-hospital |
| 101 | The Kathmandu Post | English | **140703:** https://kathmandupost.com/national/2014/07/03/woman-dies-of-snakebite; **150408:** http://kathmandupost.ekantipur.com/news/2015-04-08/govt-policy-to-blame-for-rise-in-snake-bite-deaths.html; **150901:** http://kathmandupost.ekantipur.com/news/2015-09-01/snakebites-claim-two-lives.html; **150906:** http://kathmandupost.ekantipur.com/news/2015-09-06/siblings-die-of-snakebite.html; **160628:** https://kathmandupost.com/national/2016/06/28/man-dies-due-to-snake-bite-in-kanchanpur: **170823:** http://kathmandupost.ekantipur.com/news/2017-08-23/snakebite-cases-up-in-kanchanpur.html; **180611:** https://kathmandupost.com/national/2018/06/11/teenage-girl-dies-of-snake-bite-in-chhaupadi-hut; **180703:** https://kathmandupost.com/national/2018/07/03/4-people-die-of-snakebites-in-mahottari-district; **180911:** https://kathmandupost.com/miscellaneous/2018/09/11/snake-bite-deaths-high-in-jhapa; **190627:** https://kathmandupost.com/health/2019/06/27/with-the-start-of-monsoon-number-of-snakebite-deaths-spike; **190801:** https://kathmandupost.com/health/2019/08/01/nepalis-are-dying-from-snakebites-and-it-wants-the-world-s-attention; **200420:** https://kathmandupost.com/national/2020/04/20/cases-of-snakebite-on-the-rise-with-the-onset-of-summer; **200706:** https://kathmandupost.com/sudurpaschim-province/2020/07/06/at-mahakali-hospital-patients-of-snakebite-and-covid-19-are-in-want-of-ventilator; **200727:** https://kathmandupost.com/province-no-1/2020/07/27/snakebite-victims-are-dying-in-taplejung-as-they-do-not-get-immediate-medical-attention; **210807:** https://kathmandupost.com/province-no-2/2021/08/07/bardibas-snakebite-centre-does-not-have-a-ventilator; **220214:** https://kathmandupost.com/national/2022/02/14/snakebite-an-invisible-neglected-crisis-in-nepal; **220605:** https://kathmandupost.com/national/2022/06/05/snakebite-cases-rise-with-monsoon-around-the-corner; **220703:** https://kathmandupost.com/sudurpaschim-province/2022/07/03/snakebite-patients-in-talloswarad-of-baitadi-die-for-want-of-treatment |
| 102 | The Press Nepal | Nepali | **220622:** https://thepressnepal.com/2022/06/22/36323/ |
| 103 | The Rising Nepal | English | **150627:** http://therisingnepal.org.np/news/4433; **150707:** http://therisingnepal.org.np/news/4655; **150805:** http://therisingnepal.org.np/news/6213; **220731:** https://www.risingnepaldaily.com/news/14736 |
| 104 | tikhokhabar.com | Nepali | **190905:** https://www.tikhokhabar.com/local/573#! |
| 105 | Tulsipur Online | Nepali | **190905:** https://tulsipuronline.com/bannernews/123765; **200523:** https://tulsipuronline.com/; **200717:** https://tulsipuronline.com/ |
| 106 | Tuwachung.com | Nepali | **200906:** https://www.tuwachung.com/2020/09/06/2378/ |
| 107 | unhcr.org | Nepali | **130923:** http://www.unhcr.org/524005c36.html |
| **Abbreviation:** **SN** = serial number; **URL** = uniform resource locator | | | |
