# Supplementary material for: Analysis of News Media-Reported Snakebite Envenoming in Nepal during 2010–2022
Source: PLoS Negl Trop Dis. 2023 Aug 28;17(8):e0011572. doi: 10.1371/journal.pntd.0011572 (PMC10491300; doi:10.1371/journal.pntd.0011572)
Supplement: S2 Table — (DOCX) [file pntd.0011572.s002.docx]

| **S2 Table.** Incidence of snakebites and associated envenoming and deaths reported in the news media during 2010–2022. | | | | | | | |
| --- | --- | --- | --- | --- | --- | --- | --- |
| **SN** |  | **All incidence of snakebites** | | | | | **Year ranges** (within which cases were reported) |
|  | **Categories** | Envenomings | Bites without envenoming | Undetermined snakebites | Total snakebites | Total snakebite deaths |  |
|  | **a. Districts** | | | |  |  |  |
| 1 | Achham | 4 | – | – | 4 | 4 | 2017–18, 2021–22 |
| 2 | Baglung | 1 | – | – | 1 | 1 | 2019 |
| 3 | Baitadi | 13 | – | – | 13 | 12 | 2019–22 |
| 4 | Bajhang | 2 | – | – | 2 | 2 | 2012, 2019 |
| 5 | Banke | 20 | 27 | – | 47 | 2 | 2016–18, 2020 |
| 6 | Bara | 8 | – | – | 8 | 6 | 2010, 2013–14, 2016, 2020–21 |
| 7 | Bardiya | 8 | – | – | 8 | 5 | 2012, 2015–16, 2018, 2020, 2022 |
| 8 | Bhaktapur | 2 | – | – | 2 |  | 2015 |
| 9 | Chitwan | 5 | 1 | – | 6 | 3 | 2014, 2018, 2020–21 |
| 10 | Dailekh | 2 | – | – | 2 | 2 | 2017 |
| 11 | Dang | 63 | – | – | 63 | 59 | 2015, 2017–22 |
| 12 | Dhadhing | 3 | – | – | 3 | 1 | 2016, 2022 |
| 13 | Dhanusha | 1 | – | – | 1 | 1 | 2020 |
| 14 | Dolakha | 1 | – | – | 1 | – | 2022 |
| 15 | Doti | 5 | – | – | 5 | 5 | 2015, 2019–20 |
| 16 | Gorkha | 1 | – | 1 | 2 | – | 2019 |
| 17 | Humla | 3 | – | – | 3 | 3 | 2018–19 |
| 18 | Ilam | 5 | – | – | 5 | 5 | 2011, 2017, 2019 |
| 19 | Jhapa | 225 | 800 | – | 1025 | 24 | 2013, 2015, 2016, 2018–19, 2021–22 |
| 20 | Kailali | 12 | – | – | 12 | 7 | 2010, 2013–14, 2018–19, 2020, 2022 |
| 21 | Kanchanpur | 62 | – | – | 62 | 57 | 2010–17, 2020–22 |
| 22 | Kapilvastu | 5 | – | – | 5 | 5 | 2020 |
| 23 | Kaski | 2 | – | – | 2 | – | 2020–21 |
| 24 | Kathmandu | 1 | – | – | 1 | 1 | 2016 |
| 25 | Khotang | – | 2 | – | 2 | – | 2018 |
| 26 | Lalitpur | – | – | 1 | 1 | – | 2022 |
| 27 | Lamjung | 1 | – | – | 1 | 1 | 2014 |
| 28 | Mahottari | 39 | 2 | 28 | 69 | 28 | 2015–22 |
| 29 | Makawanpur | 1 | – | – | 1 | – | 2022 |
| 30 | Morang | 12 | – | 2 | 14 | 8 | 2015, 2017–19, 2021–22 |
| 31 | Nawalpur (aka Nawalparasi East) | 5 | – | – | 5 | 5 | 2016, 2021 |
| 32 | Nuwakot | – | – | 1 | 1 | – | 2020 |
| 33 | Palpa | 2 | – | – | 2 | 2 | 2014, 2018 |
| 34 | Panchthar | 1 | – | – | 1 | – | 2017 |
| 35 | Parasi (aka Nawalparasi West) | 5 | – | 2 | 7 | 3 | 2015, 2021–22 |
| 36 | Parbat | 1 | – | – | 1 | 1 | 2020 |
| 37 | Parsa | 5 | – | – | 5 | 4 | 2015, 2020 |
| 38 | Pyuthan | 6 | – | – | 6 | 6 | 2018, 2020 |
| 39 | Rasuwa | 1 | – | – | 1 | 1 | 2015 |
| 40 | Rautahat | 23 | – | – | 23 | 11 | 2014–19, 2021 |
| 41 | Rolpa | 1 | – | – | 1 | 1 | 2022 |
| 42 | Rupandehi | 5 | – | 1 | 6 | 5 | 2014–15, 2020–22 |
| 43 | Salyan | 7 | – | – | 7 | 7 | 2017, 2022 |
| 44 | Saptari | 39 | – | 10 | 49 | 22 | 2012, 2015, 2017–22 |
| 45 | Sarlahi | 5 | – | – | 5 | 5 | 2015, 2019, 2022 |
| 46 | Sindhuli | 15 | 1 | 240 | 256 | 2 | 2014, 2018–19, 2021–22 |
| 47 | Sindhupalchowk | 1 | – | – | 1 | – | 2016 |
| 48 | Siraha | 6 | – | – | 6 | 6 | 2017–18, 2020, 2022 |
| 49 | Sunsari | 4 | – | 500 | 504 | 4 | 2010, 2015, 2019–20 |
| 50 | Surkhet | 4 | – | – | 4 | 3 | 2018, 2020, 2022 |
| 51 | Syangja | 1 | – | – | 1 | 1 | 2022 |
| 52 | Taplejung | 23 | – | – | 23 | 20 | 2012, 2015, 2018–21 |
| 53 | Udayapur | 12 | – | – | 12 | 12 | 2010, 2013, 2016, 2018–20 |
|  | ***Subtotal A*** | ***679*** | ***833*** | ***786*** | ***2298*** | ***363*** |  |
|  | **b. Undefined areas (UA)** | | | | |  |  |
| 1 | Terain areas (unspecified geography) | 5 | – | 20 | 25 | 4 | 2012, 2019 |
| 2 | Nawalparasi (either Nawalpur or Parasi District) | 1 | – | 48 | 49 | 1 | 2019, 2022 |
|  | ***Subtotal B*** | ***6*** | – | ***68*** | ***74*** | ***5*** |  |
|  | **c. Snakebite Treatment Centers** where antivenom was supplied during the report of snakebites | | | | | | |
| 1 | Banke District's Nepalgunj based Bheri Zonal Hospital (aka Bheri Hospital) Data | 906 | – | 7 | 913 | 65 | 2014–18, 2020, 2022 |
| 2 | Bara District Hospital, Kalaiya (aka Kalaiya Hospital) Data | 6 | – | 149 | 155 | 6 | 2010, 2016 |
| 3 | Bardiya District's Gulariya based Bardiya District Hospital Data | 3 | – | 54 | 57 | 1 | 2018 |
| 4 | Bardiya District's Thakurbaba Municipality, Sainbar based STC (run by Nepal Army) | 10 | – | 237 | 247 | 10 | 2014–17 |
| 5 | Chitwan District's Bharatpur based Bharatpur Hospital Data | 362 | 800 | 1496 | 2658 | 33 | 2013–14, 2017, 2019–21 |
| 6 | Dhanusha District's Kshireshwarnath Municipality-05 based STC Data | – | – | 585 | 585 | – | 2021–22 |
| 7 | Jhapa District's Charaali based STC | – | – | 4188 | 4188 | – | 2014–15, 2018–20 |
| 8 | Jhapa District's Damak based STC (run by Nepal Red Cross Society) Data | 130 | 1170 | 3226 | 4526 | – | 2017–19, 2020 |
| 9 | Jhapa District's Kankaimai Municipality-04, Kotihom based Kankai Sahara STC Data | 5 | 65 | 250 | 320 | – | 2018–19 |
| 10 | Jhapa District's Kehankawal Rural Municipality, Baniyani-based STC Data | 6 | – | 239 | 245 | – | 2021–22 |
| 11 | Kailali District's Badhaipur based STC (run by Nepal Army) Data | – | – | 771 | 771 | – | 2016–20 |
| 12 | Kailali District's Dhangadi based Seti Zonal Hospital (now aka Seti Provincial Hospital) Data | 162 | 186 | 481 | 829 | 36 | 2014, 2016–19, 2022 |
| 13 | Kanchanpur District's Arjuni based STC (operated by Nepal Army) Data | – | – | 753 | 753 | – | 2014–19 |
| 14 | Kanchanpur District's Bheemdatta Municipality, Mahendranagar based Mahakali Zonal Hospital (aka Mahakali Hospital) Data | 110 | – | 251 | 361 | 2 | 2016–17, 2020–21 |
| 15 | Kapilvastu District's Buddhabhumi-2, Garusinghe based STC Data | 2 | – | 400 | 402 | 2 | 2017, 2020 |
| 16 | Mahottari District's Bardibas-03, Gauridanda based STC Data | 72 | 1094 | 1931 | 3097 | 4 | 2014–22 |
| 17 | Mahottari District's Jaleshwor based Jaleshwor Hospital Data | 4 | 57 | 430 | 491 | 1 | 2013, 2018, 2021 |
| 18 | Morang District's Belbari-based STC (run by Nepal Army and Belbari Municipality) Data | – | – | 26 | 26 | – | 2022 |
| 19 | Morang District's Biratnagar based Koshi Zonal Hospital Data | 50 | 79 | 1050 | 1179 | 3 | 2012, 2017, 2019 |
| 20 | Nawalpur District's Binaye-Tribeni 6, Tribeni based Gorakhdal Gan STC Data | – | – | 444 | 444 | – | 2021–22 |
| 21 | Parasi District's Ramgram-05, Buddha Chock based Prithivichandra Hospital Data | – | – | 373 | 373 | – | 2015, 2019, 2022 |
| 22 | Parasi District's Sunwal NaPa 11, Jargaha based Prahariyukti Talim Mahavidhyala STC Data | – | – | 110 | 110 | – | 2021 |
| 23 | Parasi District's Bardaghat based Chisapani Hospital Data | – | – | 105 | 105 | – | 2019 |
| 24 | Parsa District's Birgunj based Narayeni Hospital Data | 4 | – | 296 | 300 | 4 | 2020 |
| 25 | Rautahat District Hospital, Gaur Data | 19 | 295 | 1140 | 1454 | 13 | 2014–17 |
| 26 | Rupandehi District's Bhairahawa based Bhim Hospital | 1155 | – | – | 1155 | – | 2017 |
| 27 | Rupandehi District's Butwal based Lumbini Zonal Hospital Data | 505 | – | 4019 | 4524 | 80 | 2011, 2013, 2016–17 |
| 28 | Saptari District's Khadak Municipality-07, Kalyanpur based STC (run by Nepl Army) Data | 10 | 204 | 352 | 566 | – | 2019 |
| 29 | Saptari District's Rajbiraj based Gajendra Narayan Singh Sagarmatha Zonal Hospital Data | 9 | – | 1136 | 1145 | 2 | 2017–20 |
| 30 | Sarlahi District's Malangawa based Sarlahi District Hospital Data | 11 | – | 150 | 161 | 11 | 2015, 2019 |
| 31 | Sarlahi District's Nawalpur based STC Data | 52 | 500 | 94 | 646 | 2 | 2015, 2019, 2021–22 |
| 32 | Shukraraj Tropical and Infectious Disease Hosptal, Teku (aka Teku Hospital) data | 7 | – | 1398 | 1405 | – | 2013–14, 2019, 2021–22 |
| 33 | Sindhuli District's Dudhauli Municipality-09, Dudhauli based STC Data | 4 | 40 | 428 | 472 | 1 | 2015–17, 2019 |
| 34 | Siraha District's Badharamal of Bandipur based Community STC (run by Nepal Army) Data | NA | – | 91 | 91 | – | 2019 |
| 35 | Siraha District's Choharwa based Jayakali STC Data | 52 | 467 | 158 | 677 | – | 2017–19 |
| 36 | Sunsari District's Itahari based Community STC Data | 191 | 238 | 3624 | 4053 | – | 2017–19 |
| 37 | Udayapur District's Katari Municipality based STC Data | 1 | – | 163 | 164 | 1 | 2018–19 |
|  | ***Subtotal C*** | ***3848*** | ***5195*** | ***30605*** | ***39648*** | ***277*** |  |
|  | **d. Hospitals where snakebite cases were accessed** (availability of antivenom to these healthcare systems was unknown) | | | | | | |
| 1 | Baitadi District Hospital Data | 7 | – | – | 7 | – | 2019 |
| 2 | Darchula District Hospital Data | 1 | – | 2 | 3 | – | 2018 |
| 3 | Jhapa District's Damak-based Amda Hospital Data | – | – | 1029 | 1029 | – | 2013 |
| 4 | Khotang District Hospital, Diktel Data | – | – | 151 | 151 | – | 2015–17 |
| 5 | Nuwakot District's Bidur based Trishuli Hospital Data | – | – | 150 | 150 | – | 2019 |
| 6 | Rasuwa District Hospital Data | 2 | – | 10 | 12 | – | 2016 |
| 7 | Tanahun District's Damauli based Damauli Hospital Data | – | – | 60 | 60 | – | 2020 |
|  | ***Subtotal D*** | ***10*** | – | ***1402*** | ***1412*** | – |  |
|  | **e. Nepal Police Offices** |  |  |  |  |  |  |
| 1 | Jhapa District Police Office Data | 2 | – | – | 2 | 2 | 2022 |
| 2 | Salyan District Police Office Data | 1 | – | – | 1 | 1 | 2022 |
| 3 | Sudurpaschim Pradesh Police Office, Dipayal data | 43 | – | – | 43 | 43 | 2018 |
| 4 | Udayapur District's Area Police Office Data | 4 | – | – | 4 | 4 | 2018 |
|  | ***Subtotal E*** | ***50*** | – | – | ***50*** | ***50*** |  |
|  | **Grand total** (subtotal A+B+C+D+E) | **4593** | **6028** | **32861** | **43482** | **695** |  |
|  | **Abbreviations: aka:** also known as; **NaPa:** Nagarpalika (i.e., Municipality); **SN:** serial number; **STCs:** Snakebite Treatment Centers | | | | | | |
