# Supplementary material for: Analysis of News Media-Reported Snakebite Envenoming in Nepal during 2010–2022
Source: PLoS Negl Trop Dis. 2023 Aug 28;17(8):e0011572. doi: 10.1371/journal.pntd.0011572 (PMC10491300; doi:10.1371/journal.pntd.0011572)
Supplement: S3 Table — (DOCX) [file pntd.0011572.s003.docx]

| **S3 Table.** Comparison of distribution of snakebite envenomings and associated deaths reported by the news media during 2010–2022 by districts (and institutions) with a published report of similar incidence from 23 districts of Terai region† of Nepal. | | | | | | |
| --- | --- | --- | --- | --- | --- | --- |
|  |  | **Incidence of envenomings reported from** | | | | |
|  | **Categories** | 50 districts during this study (Baglung and Parbat Districts' cases were provided with only incidence. So, our case reports' analyses represented only 48 districts) | | | 23 districts of Terai region in other's study† | |
|  |  |  |  |  |  |  |
| **SN** | **a. Districts*** | Envenomings | Snakebite deaths | **Year ranges** (within which cases were reported) | Snakebites (n = 166) | Deaths (n = 13) |
| 1 | Jhapa | 225 | 24 | 2013, 2015–16, 2018–19, 2021–22 | 9 | 1 |
| 2 | Dang | 63 | 59 | 2015, 2017, 2018–22 | 6 | 1 |
| 3 | Kanchanpur | 62 | 57 | 2010–17, 2020–22 | 3 | 0 |
| 4 | Mahottari | 39 | 28 | 2015–18, 2020–22 | 12 | 1 |
| 5 | Saptari | 39 | 22 | 2012, 2015, 2017–22 | 28 | 1 |
| 6 | Rautahat | 23 | 11 | 2014–19, 2021 | 0 | 0 |
| 7 | Taplejung | 23 | 20 | 2012, 2015, 2018–21 | – | – |
| 8 | Banke | 20 | 2 | 2016–18, 2020 | 6 | 0 |
| 9 | Sindhuli | 15 | 2 | 2014, 2018–19, 2021, 2022 | – | – |
| 10 | Baitadi | 13 | 12 | 2019–22 | – | – |
| 11 | Kailali | 12 | 7 | 2010, 2013–14, 2018–20, 2022 | 6 | 0 |
| 12 | Morang | 12 | 8 | 2015, 2017–19, 2021–22 | 5 | 0 |
| 13 | Udayapur | 12 | 12 | 2010, 2013, 2016, 2018–20 | 2 | 0 |
| 14 | Bara | 8 | 6 | 2010, 2013–14, 2016, 2020–21 | 0 | 0 |
| 15 | Bardiya | 8 | 5 | 2012, 2015–16, 2018, 2020, 2022 | 3 | 0 |
| 16 | Salyan | 7 | 7 | 2017, 2022 | – | – |
| 17 | Pyuthan | 6 | 6 | 2018, 2020 | – | – |
| 18 | Siraha | 6 | 6 | 2017–18, 2020, 2022 | 8 | 1 |
| 19 | Chitwan | 5 | 3 | 2014, 2018, 2020 | 3 | 1 |
| 20 | Doti | 5 | 5 | 2015, 2019–20 | – | – |
| 21 | Ilam | 5 | 5 | 2011, 2017, 2019 | – | – |
| 22 | Kapilvastu | 5 | 5 | 2020 | 12 | 1 |
| 23 | Nawalpur (aka Nawalparasi East) | 5 | 5 | 2016, 2021 | – | – |
| 24 | Parasi (aka Nawalparasi West) | 5 | 3 | 2015, 2021–22 | – | – |
| 25 | Parsa | 5 | 4 | 2015, 2020 | 1 | 0 |
| 26 | Rupandehi | 5 | 5 | 2014–15, 2020, 2022 | 12 | 0 |
| 27 | Sarlahi | 5 | 5 | 2015, 2019, 2022 | 26 | 4 |
| 28 | Achham | 4 | 4 | 2017–18, 2021–22 | – | – |
| 29 | Sunsari | 4 | 4 | 2015, 2019–20 | 6 | 0 |
| 30 | Surkhet | 4 | 3 | 2018, 2020, 2022 | 3 | 0 |
| 31 | Dhadhing | 3 | 1 | 2016, 2022 | – | – |
| 32 | Humla | 3 | 3 | 2018–19 | – | – |
| 33 | Bajhang | 2 | 2 | 2012, 2019 | – | – |
| 34 | Bhaktapur | 2 | – | 2015 | – | – |
| 35 | Dailekh | 2 | 2 | 2017 | – | – |
| 36 | Kaski | 2 | – | 2020–21 | – | – |
| 37 | Palpa | 2 | 2 | 2014, 2018 | – | – |
| 38 | Baglung | 1 | 1 | 2019 | – | – |
| 39 | Dhanusha | 1 | 1 | 2020 | 7 | 0 |
| 40 | Dolakha | 1 | – | 2022 | – | – |
| 41 | Gorkha | 1 | – | 2019 | – | – |
| 42 | Kathmandu | 1 | 1 | 2016 | – | – |
| 43 | Lamjung | 1 | 1 | 2014 | – | – |
| 44 | Makawanpur | 1 | – | 2022 | 7 | 1 |
| 45 | Panchthar | 1 | – | 2017 | – | – |
| 46 | Parbat | 1 | 1 | 2020 | – | – |
| 47 | Rasuwa | 1 | 1 | 2015 | – | – |
| 48 | Rolpa | 1 | 1 | 2022 | – | – |
| 49 | Sindhupalchowk | 1 | – | 2016 | – | – |
| 50 | Syangja | 1 | 1 | 2022 | – | – |
|  | ***Subtotal A*** | ***679*** | ***363*** |  | – | – |
|  | **b. Undefined areas (UA)** | |  |  |  |  |
| 1 | Terain areas | 5 | 4 | 2012, 2019 | – | – |
| 2 | Nawalparasi (either Nawalpur or Parasi District) | 1 | 1 | 2022 | 8 | 1 |
|  | ***Subtotal B*** | ***6*** | ***5*** |  | – | – |
|  | **c. Snakebite Treatment Centers** where antivenom was supplied during the report of snakebites by news media reporters | | | | | |
| 1 | Rupandehi District's Butwal based Lumbini Zonal Hospital Data | 505 | 80 | 2011, 2013, 2016–17 | – | – |
| 2 | Rupandehi District's Bhairahawa based Bhim Hospital | 1155 | – | 2017 | – | – |
| 3 | Banke District's Nepalgunj based Bheri Zonal Hospital (aka Bheri Hospital) | 906 | 65 | 2014, 2015–18, 2020, 2022 | – | – |
| 4 | Chitwan District's Bharatpur based Bharatpur Hospital Data | 362 | 33 | 2014, 2017, 2020, 2021 | – | – |
| 5 | Sunsari District's Itahari based Community STC Data | 191 | – | 2017–18 | – | – |
| 6 | Kailali District's Dhangadi based Seti Zonal Hospital (now aka Seti Provincial Hospital) Data | 162 | 36 | 2014, 2016–18, 2022 | – | – |
| 7 | Jhapa District's Damak based STC (run by Nepal Red Cross Society) Data | 130 | – | 2018 | – | – |
| 8 | Kanchanpur District's Bheemdatta Municipality, Mahendranagar based Mahakali Zonal Hospital (aka Mahakali Hospital) Data | 110 | 2 | 2016–17, 2020–21 | – | – |
| 9 | Mahottari District's Bardibas-03, Gauridanda based STC Data | 72 | 4 | 2014–17, 2019–22 | – | – |
| 10 | Sarlahi District's Nawalpur based STC Data | 52 | 2 | 2021–22 | – | – |
| 11 | Siraha District's Choharwa based Jayakali STC | 52 | – | 2017–18 | – | – |
| 12 | Morang District's, Biratnagar based Koshi Zonal Hospital Data | 50 | 3 | 2012 | – | – |
| 13 | Rautahat District Hospital, Gaur Data | 19 | 13 | 2014–17 | – | – |
| 14 | Sarlahi District's Malangawa based Sarlahi District Hospital Data | 11 | 11 | 2019 | – | – |
| 15 | Bardiya District's Thakurbaba Municipality, Sainbar based STC (run by Nepal Army) | 10 | 10 | 2017 | – | – |
| 16 | Saptari District's Khadak Municipality-07, Kalyanpur based STC (run by Nepl Army) Data | 10 | – | 2019 | – | – |
| 17 | Saptari District's Rajbiraj based Gajendra Narayan Singh Sagarmatha Zonal Hospital Data | 9 | 2 | 2019 | – | – |
| 18 | Shukraraj Tropical and Infectious Disease Hosptal, Teku (aka Teku Hospital) Data | 7 | – | 2019 | – | – |
| 19 | Bara District Hospital, Kalaiya (aka Kalaiya Hospital) Data | 6 | 6 | 2010 | – | – |
| 20 | Jhapa District's Kehankawal Rural Municipality, Baniyani-based STC Data | 6 | – | 2021 | – | – |
| 21 | Jhapa District's Kankaimai Municipality-04, Kotihom based Kankai Sahara STC Data | 5 | – | 2019 | – | – |
| 22 | Mahottari District's Jaleshwor based Jaleshwor Hospital Data | 4 | 1 | 2013 | – | – |
| 23 | Parsa District's Birgunj based Narayeni Hospital Data | 4 | 4 | 2020 | – | – |
| 24 | Sindhuli District's Dudhauli Municipality-09, Dudhauli based STC Data | 4 | 1 | 2016–17 | – | – |
| 25 | Bardiya District's Gulariya based Bardiya District Hospital Data | 3 | 1 | 2018 | – | – |
| 26 | Kapilvastu District's Buddhabhumi-02, Garusinghe based STC Data | 2 | 2 | 2020 | – | – |
| 27 | Udayapur District's Katari Municipality based STC Data | 1 | 1 | 2018 | – | – |
|  | ***Subtotal C*** | ***3848*** | ***277*** |  | – | – |
|  | **d. Known from hospitals where snakebite cases accessed** (availability of antivenom to these healthcare systems was unknown) | | | |  |  |
| 1 | Baitadi District Hospital Data | 7 | – | 2019 | – | – |
| 2 | Rasuwa District Hospital Data | 2 | – | 2016 | – | – |
| 3 | Darchula District Hospital Data | 1 | – | 2018 | – | – |
|  | ***Subtotal D*** | ***10*** | ***–*** |  | – | – |
|  | **e. Nepal Police Offices** |  |  |  |  |  |
| 1 | Sudurpaschim Pradesh Police Office, Dipayal Data | 43 | 43 | 2018 | – | – |
| 2 | Udayapur District's Area Police Office Data | 4 | 4 | 2018 | – | – |
| 3 | Jhapa District Police Office Data | 2 | 2 | 2022 | – | – |
| 4 | Salyan District Police Office Data | 1 | 1 | 2022 | – | – |
|  | ***Subtotal E*** | ***50*** | ***50*** |  | – | – |
|  | **Grand total** (subtotal A+B+C+D+E) | **4593** | **695** |  |  |  |
|  | **Abbreviations and symbols: aka:** also known as; **SN:** serial number; **STCs:** Snakebite Treatment Centers; *****According to Nepal Population Census 2021, total population of Nepal is 29192480 and total population of 50 districts mentioned above is 24785096 i.e., 85% of national population (with males: 12141765, 49%; females: 12643331, 51%); **†**Snakebite epidemiology in humans and domestic animals across the Terai region in Nepal: a multicluster random survey. Lancet Global Health, 10 (3): e398-e408. | | | | | |
