# Supplementary material for: Analysis of News Media-Reported Snakebite Envenoming in Nepal during 2010–2022
Source: PLoS Negl Trop Dis. 2023 Aug 28;17(8):e0011572. doi: 10.1371/journal.pntd.0011572 (PMC10491300; doi:10.1371/journal.pntd.0011572)
Supplement: Attachment — Submitted filename: Analytical tables should be added.docx [file pntd.0011572.s005.docx]

| **Table Crosstab** | | | | | | | |  |
| --- | --- | --- | --- | --- | --- | --- | --- | --- |
|  | **p-value  chi-square** | |  | |  | | |  |
|  | | * | | )%)n | |  | ***Sex*** | |
|  |  |  |  | )%)n | |  |  |  |
|  |  |  |  | **n(100%)** | | **Total** |  |  |
|  |  | * | | )%)n | |  | ***Age-classes*** | |
|  |  |  |  | )%)n | |  |  |  |
|  |  |  |  | )%)n | |  |  |  |
|  |  |  |  | **n(100%)** | | **Total** |  |  |
|  | | You should use the chi-square test here  Use the good fit test and an assignment on the hypothesis Are injuries distributed evenly between ages? Are the injuries distributed evenly between the sex?  also for Occupation And another variable you can do it. | | | | | | |

|  | | **Table Crosstab** | | | | | |
| --- | --- | --- | --- | --- | --- | --- | --- |
| **p-value  chi-square** | | **Total** | **Groups of Human habitations** | |  | | |
|  |  |  | **In** | Out |  |  |  |
| * | **n(100%)** | | )%)n | )%)n | |  | **Features num.1**  Time |
|  | **n(100%)** | | )%)n | )%)n | |  |  |
|  | **n(100%)** | | )%)n | )%)n | | **Total** |  |
| * | **n(100%)** | | )%)n | )%)n | |  | **Features num.2**  Seasons |
|  | **n(100%)** | | )%)n | )%)n | |  |  |
|  | **n(100%)** | | )%)n | )%)n | |  |  |
|  | **n(100%)** | | )%)n | )%)n | | **Total** |  |
| You should use the chi-square test here  Use the independence test and test the hypothesis. Do  **Human habitations** have a relationship with the time of injury?  Don't just list the numbers without making it clear that there is a relationship by interpreting the p-value  Do the same for another variable and for the Activeness of victims , Active / Passive | | | | | | | |
