# Supplementary material for: Analysis of News Media-Reported Snakebite Envenoming in Nepal during 2010–2022
Source: PLoS Negl Trop Dis. 2023 Aug 28;17(8):e0011572. doi: 10.1371/journal.pntd.0011572 (PMC10491300; doi:10.1371/journal.pntd.0011572)
Supplement: Attachment — Submitted filename: Authors responses_to_reviewers_R0_230731.docx [file pntd.0011572.s006.docx]

Date: Jul 10 2023 11:22AM

To: "Deb Prasad PANDEY" debpandey@gmail.com

cc: "Narayan B Thapa" drnarayanthapa@gmail.com

From: "PLOS Neglected Tropical Diseases" plosntds@plos.org

Subject: Decision on your PLOS Neglected Tropical Diseases submission (PNTD-D-23-00747)

Attachment(s): Analytical tables should be added.docx

Dear Dr. PANDEY,

Thank you very much for submitting your manuscript "Analysis of News Media-Reported Snakebite Envenoming in Nepal during 2010–2022" for consideration at PLOS Neglected Tropical Diseases. As with all papers reviewed by the journal, your manuscript was reviewed by members of the editorial board and by several independent reviewers. In light of the reviews (below this email), we would like to invite the resubmission of a significantly-revised version that takes into account the reviewers' comments.

We cannot make any decision about publication until we have seen the revised manuscript and your response to the reviewers' comments. Your revised manuscript is also likely to be sent to reviewers for further evaluation.

When you are ready to resubmit, please upload the following:

[1] **A letter containing a detailed list of your responses to the review comments** and a description of the changes you have made in the manuscript. Please note while forming your response, if your article is accepted, you may have the opportunity to make the peer review history publicly available. The record will include editor decision letters (with reviews) and your responses to reviewer comments. If eligible, we will contact you to opt in or out.

[2] Two versions of the revised manuscript: one with either highlights or **tracked changes** denoting where the text has been changed; the other a **clean version** (uploaded as the manuscript file).

Important additional instructions are given below your reviewer comments.

Please prepare and submit your revised manuscript within 60 days. If you anticipate any delay, please let us know the expected resubmission date by replying to this email. Please note that revised manuscripts received after the 60-day due date may require evaluation and peer review similar to newly submitted manuscripts.

Thank you again for your submission. We hope that our editorial process has been constructive so far, and we welcome your feedback at any time. Please don't hesitate to contact us if you have any questions or comments.

Sincerely,

Wuelton M. Monteiro, Ph.D.

Section Editor

PLOS Neglected Tropical Diseases

Wuelton Monteiro

Section Editor

PLOS Neglected Tropical Diseases

***********************

######################

Reviewer's Responses to Questions

Key Review Criteria Required for Acceptance?

As you describe the new analyses required for acceptance, please consider the following:

# Methods

1-Are the objectives of the study clearly articulated with a clear testable hypothesis stated?

2-Is the study design appropriate to address the stated objectives?

3-Is the population clearly described and appropriate for the hypothesis being tested?

4-Is the sample size sufficient to ensure adequate power to address the hypothesis being tested?

5-Were correct statistical analysis used to support conclusions?

6-Are there concerns about ethical or regulatory requirements being met?

## Reviewer #1:

The study design was appropriate to achieve the proposed objective.

The approach in which this article addresses the epidemiology of venomous snake occurs in Nepal is one of its primary contributions.

The authors conducted a comprehensive evaluation of data from reports and mapped reported cases across the country. This provided an in-depth comprehension of the geographical distribution of venomous snake bites and the demographic characteristics of the victims. The finding that children are the most vulnerable group is alarming, but it also highlights the significance of focusing preventive efforts on this age group. In addition, this methodology can be applied to additional contexts and countries in future research. Especially in countries where the government has no interest in registering cases of snake venom poisoning.

**Authors:** Dear Reviewer#1, thank you for your critical thinking and assessment regarding the methods and impact of this cost-effective approach of evaluating situation and burden of snakebite envenomation particularly in the resource-poor nations/communities.

## Reviewer #2:

1. NO, IT IS JUST DESCRIPTIVE. THERE IS NO STATISTICAL ANALYSIS AND **#^1^**TEST HYPOTHESIS AT ALL.

2. Yes, But there is a **#^2^**need for improvement.

how do the authors deal with the Data to achieve stated objectives?

what they do as descriptive is not enough to address the stated objectives.

but If the authors have a point of view about the research objectives How was it achieved? Please Explain that.

3. Yes, the population is clearly described But there are **#^3^**no hypotheses being tested.

4. No. Please provide more details, and Explain that.

**#^4^**Did the authors calculate the sample size?

The size of the sample is 296 published media news. What about the unpublished snakebite incidents?

Which can be registered with hospitals or another way? How was it taken into account?

The sample size is 296, but during research paragraphs, you used several other numbers!!

such as the number of envenoming cases=308

How do you justify that the number of envenoming cases is greater than the sample size?

Why did the sample not adopt the number of envenoming cases instead of the published news?

When calculating the ratios, it was not clear that they belong to the number of envenoming cases or to the number of published news In addition to your use of Figure No. 2, the sample size = 533

Please specify precisely and clearly a fixed sample size.

5. No. the authors did not use **#^5^**statistical analysis tests. and here is how they should be improved:

A. Enriching the research with statistical tests Not just descriptive narration numbers and percentages. So Each of the following paragraphs requires at least one statistical test for the results to be significant.

Line 260 i) Demographics

Line 275 a) Locality of snakebites

Line 285 b) Places and victims' activities when bitten

Line 293 c) Bitten body parts

Line 299 d) Temporal patterns of snakebite envenomings

Line 304 e) Snakes responsible for bites

Line 313 (DOH**#^7^**) and treatment-seeking behavior

B. **#^6^**Determining the factors affecting snakebite incidents through chi-square tests that fit the data (independence test and good fitness test), which factors are more influential in the geographical location? The time of the accident? Characteristics of the victim? Age, gender, or others, so multivariate analysis can be used and constitute an added value to the research results.

C. Use the **#^7^**Mean ± SD to describe numeric data, You Used median and IQR Explain Why?

D. I strongly **#^8^**recommend using percentages and the number next to each other, indicating the percentage to whom it belongs, for example, lines 27 - 34. Put the percentages next to each number mentioned and specify whether the percentage is from 296 or is it the percentage from 308.

Some paragraphs shifted the number and proportions to this, but some paragraphs did not. Explain that

6. **#^9^**NO.

**Authors:** Thank you Reviewer#2 for your critical reviews.

**#^1^**We improved objectives of this study with a clear testable hypothesis.

**#^2^**We improved the method section to meet with the stated objectives.

**#^3^** We further defined the study population and tested hypothesis. Accordingly, we improved methods, results, discussion, and conclusion sections.

**#^4^**As suggested you and reviewer 3, we considered our process of determining eligible samples (i.e., 308 envenomed cases herein) to be a random sampling. To estimate the appropriate sample size (i.e., the expected size of envenomings from the defined population of this study), we used Yamane formula.

In this Yamane formula, n = sample size, N = population size, d = margin of error **(**Yamane, Taro. (1967). Statistics: An Introductory Analysis, 2nd Edition, New York: Harper and Row). The details of sample size is described in the second paragraph of "Data sources and sample size" subsection of the Method section.

Our estimated sample size was 297. But, we included all eligible samples (i.e., 308 envenomed cases, Figure 2). This sample size is sufficient to address the hypothesis being tested.

To find 199 eligible articles, we accessed 533 news media articles. Those articles were collected randomly using specific keys (mentioned in method section in detail). From among the eligible 199 articles, we counted 308 envenomed cases with substantial information. There was possibility of reporting more than two snakebite case reports in a single journal article. Therefore, the sample size 308 was greater than the above mentioned first and second screened article numbers (i.e., 296 and 199, respectively).

The figure 296 was not our sample size. After the first screening of articles from 533 articles which we initially accessed, we included 296 articles. Therefore, the 296 was articles' number. In the second screening, we excluded 97 articles from among 296 articles and obtained 199 eligible articles which we pored over to extract the information for this study.

**#^5^**We improved statistical analysis involving hypothesis tests and improved Demographics, Locality of snakebites, Places and victims' activities when bitten, Bitten body parts, Temporal patterns of snakebite envenomings, Snakes responsible for bites, and DOH and treatment-seeking behavior subsections including significant findings of statistical tests.

**#^6^**Considering overall sample size and nature of data, we preferred "Multinomial logistic regression. But, our outcome variable (e.g., outcomes of envenomings in particular) has empty cells or small cells. Same applies for some predictor variables. We found such empty or small cells while cross-checking for empty or small cells in cross-tabulation between categorical predictors and the outcome variable. So, the model become unstable in some cases and did not even run at all when there were empty cells. Therefore, we used Chi-square tests to know the possible association between variables mentioned in Table 3 and 4.

**#^7^**Mean ± SD to describe numeric data, You Used median and IQR Explain Why?: Our continuous data ("age", "DOH", and "LOD") for 308 envenomated cases with substantial information were not normally distributed. If they were normally distributed we would use Mean ± SD and t-test. Because of non-normality of data distribution, we presented these continuous data as medians, interquartile ranges (IQR), and ranges. To measure 95% confidence interval (CI) of age, DOH, and LOD, we used non-parametric two-tailed Wilcoxon signed rank test with continuity correction. All about the normality and statistical measurements are mentioned in 6th paragraph of Data analysis subsection of Method section. Wilcoxon test is one of the commonly used nonparametric alternatives for the most commonly used parametric hypothesis tests.

Our samples represented diverse locations from lowlands to the hills where different snake species distribute. For example, distribution and density of krait species (nocturnal species) are greater in Terai where they intrude often into residential areas causing snakebite of sleeping people. Next, distribution and diversity of pitvipers is greater in the hills compared to Terai where they cause bite of people active in agrarian areas or forested areas. In this way, our samples represented different locations and different venomous snake species bites. This might have caused non-normality. So, we used nonparametric alternatives for parametric tests.

**#^8^**recommend using percentages and the number next to each other:

We improved this section of abstract as "We analyzed 308 Nepalese snakebite envenomed cases reported in 199 news media articles published between 2010 and 2022 to know why and how victims were bitten, their treatment-seeking behavior, and the outcomes. These envenomed cases known with substantial information represented 48 districts.". Now, it should be clearer. Further, in the data analysis section, we added about the use of percentages next to each of given number of patients. In this section, we specified about the percentage was calculated from the number of total eligible envenomed cases (i.e., 308), not from the article numbers (i.e., 533, 296, and 199). Following complete case analysis, we excluded missing values (i.e., NA). This reduced the cases from a total of 308 envenomed cases while analysing percentage in some cases.

**#^9^**Thank you.

## Reviewer #3:

The strength of this paper is it clearly explains the objectives of the study the way it has written is that anyone who can read the paper can be easily understand it. However it is better to include the **^#1^statement of the hypothesis clearly to be tested** in order to achieve the objectives of the study in the paper.

The inclusion and exclusion of the data in the paper is good and clearly understandable for any readers who were out of specialization.

The study design for this paper of analysis is an clear and attractive. I am looking here is that in the method of data analysis, **^#2^**in search of traditional snakebite healers, there is no clear way which variable is collected from this source of data ( I mean whether they have conduct an interview or they have used publish documents).

In addition in this source it is not clear **^#3^**how to determine the species of snakes.

In the methodology part states that the **^#4^**continuous variable is normally distributed and median, range and inter-quartile range is used for the analysis. If there is a continuous variable arithmetic mean, variance, standard deviation, minimum and maximum values are appropriate to describe the output of the result.

The population that have defined in the paper is clearly described and appropriate for the research objectives. The **^#1^**sample size in the paper for which organized is so much enough to made adequate conclusion or decisions for the hypothesis.

The sample is already taken from the media reports; there is no any procedure to select the sample from the population. Hence it is adequate to test the hypothesis of the research objectives.

The **^#1^**statistical analysis is good to support the conclusion. But you have not test the normality of the variable.

Since the organization of the paper uses online public data, there is no ethical concern.

**Authors:** Thank you Reviewer#3 for your critical review comments. We have responded to your concerns indicating with superscripts of #1, #2, ... etc. below.

^#1^statement of the hypothesis clearly to be tested in order to achieve the objectives of the study in the paper. ....The ^#1^sample size in the paper for which organized is so much enough to made adequate conclusion or decisions for the hypothesis...:

As it is suggested by reviewer #3 (herein) and reviewer#2 (above, response#4), we determined sample size which is enough to hypothesis testing. Accordingly, we tested hypothesis of association using Chi-Squared test. We briefly mentioned the extension of the objectives (i.e., testing of any associations, example, association between male and female victims, and other categorical variables) in the Introduction section. The respective findings are mentioned in the Result section. Accordingly, we improved Discussion and Conclusion sections. Also, we slightly improved Abstract as well.

**^#2^**in search of traditional snakebite healers: In addition to searching data in other occasions as mentioned in the "Data sources" of the Methods section, we also searched these data while listing traditional snakebite healers for a study. This search of traditional snakebite healers is just mentioned to clarify data searching strategy. If case reports mentioned in news describe about the patients seeking traditional healers, we analyzed these data under the variable "treatment seeking behavior of the patients". For this study, we did not interview these traditional healers. We referred the published reference because we preferred to make readers of this article aware about the data searching methods wholly.

**^#3^**how to determine the species of snakes: We mentioned about the snake identification in the fourth paragraph of "Data analysis" subsection of "Methods section". We expected journalists to correctly identify the snake based on photographs of the responsible snakes taken by victims or their family members or dead snakes brought to healthcare facilities by consulting with snake experts. Even in my personal experiences, several journalists and medical officers involved in snakebite treatment used to send me involved snake's photo for the confirmed identification. It is because I am one of the snake expert as well for this country. If news media reported case reports mentioned local and/or English name, we confirmed their Generic or species name following the most used snake guidebooks in Nepal.

**^#4^** In the second last paragraph of the Data analysis subsection of Methods section, we have previously mentioned that continuous data for those envenomated cases with substantial information were not normally distributed. If there was a continuous variable, we would calculate arithmetic mean, variance, and standard deviation as well. We have mentioned ranges (i.e., minimum and maximum values for those variables). Thank you for the opportunity to clarify your important queries.

We analyzed non-normally distributed continuous variables for median, range and	inter-quartile range because normally distributed continuous variable are analyzed for	arithmetic mean, variance, standard deviation including minimum and maximum	values in order to describe the output of the results.

Considering the need of knowing location of expected mean, we have provided 95%	confidence interval using non-parametric statistical analysis.

A) Histogram of Age_230717

B) Barplot

## Reviewer #4:

The study's main objectives have been outlined, but there is a need for clarity on some of the study variables.

Specifically, in section 217, "Circumstances"**^#1^** is categorised as occurring inside or outside human habitations. However, previous statements in sections 181-182 indicate that snake bites' time, month, and year should also be considered under the "Circumstances." While the study results include some of this information, clearly stating the objectives would make it easier for readers to understand.

The study needed more clarity in **^#2^**defining the population regarding district borders (81) and stating the population count of inhabitants in the area but rather provided information on the entire area's climatic changes (120-124).

The sample size was sufficient, although it is important to note that the age grouping should be systematically organised to **^#3^**ensure regular age intervals (214).

This study retrospectively analysed existing public data and did not need ethical approval (117). The study included hospital-reported cases to gather information about treatment outcomes, which was not disclosed as a **^#4^**data source (160-168).

Additionally, using **^#5^**patients' names was mentioned at 189, raising concerns about **^#5^**data protection that should be addressed ethically (200-203).

In order to ensure the study is replicable, it is important to mention the specific **^#6^**news outlets from which the data was sourced. The attached PRISMA flow diagram did not provide this information, even though the article states that data was obtained from Google, Newspapers, Facebook, and other sources.

It is important to clarify **^#4^**which data came from which sources. While the study mentioned accessing data from treatment centres and Google, according to pages 566-567, it primarily came from newspapers, indicating a contradiction.

The **^#7^**data analysis was attributed to Microsoft Excel, but further reading reveals that data was also analysed using Google Earth Pro and R statistical programs (213 and 249).

**Authors:** Thank you Reviewer#4 for critical review comments. We have improved the manuscript following your comments/suggestions.

**^#1^**We improved "Circumstances" in Methods and Discussion sections. We placed the DOH under "pre-hospital intervention" in Method section and described the DOH under the heading "Pre-hospital intervention and treatment-seeking behavior in discussion section.

In "Data collection and management" of Methods, it appears as:

"...prehospital intervention (the duration to reach the STCs/hospital [DOH]) and	treatment-seeking behavior (i.e., carried to TSHs and/or healthcare facilities),..."

In "Data analysis" of Methods, it appears as:

"... We analyzed pre-hospital interventions by assessing time to hospital arrival	[DOH was converted into hour (h)] and those..."

In the Discussion section, the subheading appears as "iii) Consequences of prehospital	intervention (DOH) and treatment-seeking behavior. And, the first line of this	subheading is slightly improved by highlighting the median value for DOH as	"Delayed hospital admission requiring a median of 3.3 h is still a challenge for	snakebite management in Nepal.".

We hope that the presentation of circumstances, DOH and treatment seeking behavior	is consistent and readers friendly now.

**^#2^**We defined the population of 48 districts from where case reports of envenomings due to venomous snakebites were described (The parenthesis followed by district names in Table 1 was human population of the respective districts published by Nepal Population Census 2021). The added texts in the Table 1 are indicated with blue texts in the track change version of the manuscript. The human population status has been briefly mentioned in the last line of the first paragraph of subheading "Study site" in Methods section.

**^#3^**To ensure regular age intervals, we added a figure (Figure 3). In this figure, we have presented age intervals by 5 year (Figure 3. bottom) so that it could be easier to evaluate the high risk group by age. Next, we added boxplot (Figure 3. top) which would be superior to the barplot because boxplot summarizes the age structure of the envenomomed patients including outliers (if any). Therefore, we have developed boxplot for the better illustration of the patients with known age.

The need of a bar plot with regular age intervals are replaced by the line graph (Figure 3. bottom). The barplot would show only frequency data of explanatory variables listed in x-axis. The line graphs in "Figure 3. bottom" shows trend of age by regular age interval of all envenomed patients and by sex.

**^#4^**data source: Thank you for your comments on data sources. We added a sentence as "These data were primarily based on hospitals/snakebite treatment centers and police offices in Nepal." Journalist acknowledged hospitals/snakebite treatment centers' authorities or Nepal Police Offices for the case report which they mentioned in the news media that are listed in the Supplementary Table 1. We searched that news at google.com using specific keys (details are already mentioned in subsection Data Sources in the Method Section.)

**^#5^**Patients' names and data protection: We carried Ethics statement section in the last of Methods section. And, we added following texts "Although available name of patients and known geographic locations were entered into our database, we used these information to cross-check and avoid duplications. The known geographic locations where the envenomings occurred were used to generate the approximate coordinates of localities and identify rural and urban areas. Considering ethics, we did not mention patients' name and their addresses where snakebite occurred elsewhere." by considering critical review comments of reviewer#4.

**^#6^**News outlets: As you suggested, we previously mentioned the specific news outlets from which the data was sourced in the Supplementary Table 1. The flow diagram has provided this information as 107 news outlets were selected for this analysis. Now, we added the text "please, see Supplementary Table 1" for the further clearance to the readers.

**^#7^**Data analysis: Thank you for your next critical comments. We improved this section accordingly as "We performed all analyses with the R statistical program (R version 4.1.2 (2021-11-01), The R Foundation for Statistical Computing Platform), the Microsoft Excel, and the Google Earth Pro.".

## Reviewer #5:

Accept

**Authors:** Thank you Reviewer#5.

--------------------

**Results**

1-Does the analysis presented match the analysis plan?

2-Are the results clearly and completely presented?

3-Are the figures (Tables, Images) of sufficient quality for clarity?

## Reviewer #1:

The results presented by the authors corroborate to achieve the objective proposed in the manuscript.

It is also possible to verify which population presents the highest risk, which snake species is the major cause of accidents, and the circumstances of the ophidian accident.

**Authors:** Thank you Reviewer#1.

## Reviewer #2:

1. NO, IT IS JUST DESCRIPTIVE. THERE IS NO **#^1^**STATISTICAL ANALYSIS AT ALL.

2. As descriptive, yes. but there is a need for improvement and here is how they should be improved:

THE ANALYSIS RESULTS WE CAN NOT DEPEND ON WITHOUT **#^1^**SIGNIFICANT STATISTICAL TEST WITH P-VALUE.

Note that: Based on your research data, if the authors say that the percentage of envenomated women is higher than that of men, Because women are 160 (52%) and men are 144 (47%).

This is not considered a result of scientific research, it is only considered a descriptive statistic from a sample, because the difference between the ratios by 5% has not been tested statistically, So this difference may be NOT significant, until it is verified by testing. (you **#^2^**must use Chi-square test). However, the authors dealt with it and other descriptive statistics as proven scientific results, and this is a grave mistake.

3. The graph and maps are good and have an important clearance for all the information.

Is it possible to choose a **#^3^**higher resolution? For clarity without pixelation

**Authors:** Thank you Reviewer#2.

**#^1^**STATISTICAL ANALYSIS: We improved the data analyses including hypothesis testing. Along with the findings from descriptive statistics, we also included p-value of for appropriate statistical testing.

**#^2^**We realized your advice. Accordingly, we have improved our results involving Chi-square tests for goodness of fit and for independence.

**#^3^**We produced figures at minimum of 300 pixel per inch. We hope that resolutions for the figures should match with the need of journal.

## Reviewer #3:

The analysis presented in the paper very good and it is perfectly conceited with the analysis plan settled in the organization of this paper. But one variables of interest has been missed in the analysis (**^#1^**occupation of snakebite)

The result of the analysis is clearly presented. In almost all it is good and clear for any reader from other specialization to understand it. However, **^#2^**Not Applicable (NA) data values must be replaced by appropriate values. Since NA values are missing data values, it is better to **treat missing data values with appropriate missing data treatment methods**.

Even if the result for the analysis of the paper is almost all completed for all variables described in the methodology and its way of presentation is good, for the variable **^#1^**occupation of snakebite cases (farmer, student,. . .) is not listed in the result section.

In the result section, **^#3^**age and hours must be described in terms of average, standard deviation and variance instead of median, range and inter-quartile range.

I have given a great credit for the way of presenting figures and images put under the appendix part for the output. The output of figures (tables and images) displayed in the appendix part of the results for the analysis data is clear and sufficient for the describing the output of the analysis for each variable of interest. The quality of tables and images are enough to get clarity for the results describe in the result section. Abbreviations under subscript of each table have been clearly defined. Generally it is good, clear and sufficient.

**Authors:** Thank you Reviewer#3.

**^#1^**occupation of snakebite: We previously mentioned it in the last line of the last paragraph of Demographics subsection in the Results section. The details of the occupations are mentioned in the Table 2, too. For the visibility of the readers, we have placed Occupation section into a separate paragraph (the last paragraph of subsection Demographics).

**^#^**^2^Treat missing data values with appropriate missing data treatment methods: In our dataset, the missing of values were completely at random and unrelated to any of the variables involved in our analysis. Therefore, we adopted a complete case analysis [1]. This was mentioned in the 7th paragraph of the Data analysis subsection of Method section.

**^#3^**age and hours must be described in terms of average, standard deviation and variance:

Our samples represented diverse locations from lowlands to the hills where different snake species distribute. For example, distribution and density of krait species (nocturnal species) are greater in Terai where they intrude often into residential areas causing snakebite of sleeping people. Next, distribution and diversity of pitvipers is greater in the hills compared to Terai where they cause bite of people active in agrarian areas or forested areas. In this way, our samples represented different locations and different venomous snake species bites. This might have caused non-normality. So, we used nonparametric alternatives for parametric tests.

For the normal distribution of data set, mean and standard deviation/variance would be a target and for the non-normal data set, median and inter-quartile range would give the ideas on nature of data distribution. We have also analyzed interquartile range (IQR) which is more useful for describing median based variables (herein, age and hours).

Concluding above, for the normal distributions, we use the standard deviation to determine the percentage of observations that fall at specific distances from the mean. However, that doesn’t work for non-normal (skewed) distributions. Therefore, the IQR is an excellent alternative to standard deviation. We used IQR to detect any outliers in our data set following the formula mentioned below.

Q1 − 1.5 * IQR: Lower outlier gate.

Q3 + 1.5 * IQR: Upper outlier gate.

We used ranges demonstrating the minimum and maximum values which could help to decide on occurrence of any outliers.

Our data for the variable "age" were within the lower and upper outlier gate (i.e., there was no influence by outliers in age despite their skewed distribution). But, hours related data were influenced by outliers.

Additionally, like the IQR, the median is superb for skewed distributions. Therefore, considering nature of data distributions of these variables, we used median instead of mean and IQR instead of standard deviation.

Further, we calculated 95% confidence interval using the non-parametric statistical analysis using median (instead of mean) to know the range within which expected population median would be placed.

## Reviewer #4:

The study's main objectives were achieved in the results, but some modifications are necessary to ensure the article is well organised and flows smoothly.

**Authors:** Thank you. We improved this manuscript greatly. We think that this article is now better organized than the previous version. Now, it flows smoothly.

## Reviewer #5:

The results are not clearly and completely presented. Because at line 260 (Demographics) through to 264 the statement made doesn't correspond to the table 2. It was stated that "We determined that 262 sex was known in 304 cases but without age in 59 cases". So the issue here is the 59 cases without age. Where from it and if you can explain it better or make corrections.

**Authors:** Thank you for your critical review comments. We improved this section.

--------------------

# Conclusions

1-Are the conclusions supported by the data presented?

2-Are the limitations of analysis clearly described?

3-Do the authors discuss how these data can be helpful to advance our understanding of the topic under study?

4-Is public health relevance addressed?

## Reviewer #1:

This study proposes methods for mitigating the effects of venomous snake injuries. Using the collected epidemiological data, the researchers identified high-risk regions and established a firm foundation for the development of prevention and intervention programs.

One suggestion: The authors could expand the discussion on ways to mitigate the occurrence of snakebites, such as the implementation of educational measures, such as awareness campaigns and first aid training. This can play a vital role in reducing the number of fatalities resulting from venomous snake bites.

**Authors:** Thank you. We improved the last paragraph of iii^rd^ section of the Discussion as "Mass awareness campaigns and trainings on proper prehospital care are essential to ensure immediate access to a snakebite treatment centre in areas where people mostly rely on TSHs for snakebite care. This approach can diminish the dependency on TSHs, help to standardize the health of rural populations [2], and can play a vital role in reducing the number of fatalities resulting from venomous snakebites."

## Reviewer #2:

1. NO, All conclusions are based on descriptive numbers, and this is not sufficient to be considered a conclusion, because the relationships and differences assumed by the authors may not be statistically significant unless this is proven.

**Authors:** Thank you. We improved it as mentioned above.

2. Yes, there is no need for improvement.

**Authors:** Thank you.

3. Yes. but there is a need for improvement. Because The Scientific research should not be just collecting, describing and summarizing news, because in this case it will become a news publication, but not a scientific research.

**Authors:** Thank you. We improved discussion demonstrating how our findings can be helpful to advance understanding snakebite problems and its cost-effective management, and need of additional research.

4. Yes, there is no need for improvement.

**Authors:** Thank you.

## Reviewer #3:

The way of the conclusion in the paper is very good and tries to depend on the data presented in the result section. The **^#1^**conclusion must depend on all results obtained in the analysed data. But not generalize based on all variables of findings. It has concluded by only few study finding results or concluded by only few variables.

In the organization of the paper the limitation has been listed down. In the limitation part is listed only the unavailable data for the media report reports the cases were under the treatment condition. However, in the data analysis part you have described the inclusion and exclusion criteria. Under media reports some information in the cases **^#2^**may be missed like age or age groups, sex . . . These are the limitations in in conducting this paper. It is better to include all important sources which gives power for the quality of paper but not unable to access it.

This paper discussed on the understandings of snakebite envenoming cases in the study area regarding to demographic, circumstance of snakebite and outcome of bite causes. This paper show an important figure is that how traditional religious-cultural effect on girls and women in the selected study area. The authors have discussed his findings with other authors that have been given primary attention in paper organization. The way how to discuss his findings with another author’s finding has given an advance uses of understandings of the topic in the study area.

From this paper the public health relevance related to the rescue of snakebite envenoming in the study area has been addressed. The authors have fond or determine the categories of society which have been exposed to the topic of the study. Therefor any concerned body can take an action or intervention by understanding this finding in order to protect the society.

**Authors:** Thank you for your critical review. We have responded your further queries below indicating with #1 and #2, respectively:

**^#1^**conclusion must depend on all results obtained in the analysed data: We improved conclusion section including all results obtained in the analysed data.

**^#2^**may be missed like age or age groups, sex . . . These are the limitations in in conducting this paper. It is better to include all important sources which give power:

To increase the power of this paper, we attempted to find as many news as possible that are available online and print versions, too. We used different keys to get these articles. Further, we pored over even the duplicated news published in different media to find any additional data on age, age group, sex, etc. If they provided additional news, we included those news articles even if the majority of information was same. The details are mentioned in "Data sources and sample size" subsection of Methods section.

## Reviewer #4:

Carefully analysed data support the study's conclusions, and the study's limitations are clearly stated. The study's benefits are outlined and are especially relevant to the Nepalese, whose daily lives and cultural practices often involve exposure to venomous snakes. While the study successfully answers the questions it raises and achieves its objectives, these objectives were scattered throughout the article instead of being clearly stated in the introduction, which may make it difficult for readers to understand the study's main goals, as noted in section 124.

**Authors:** Thank you for your critical review comments. Following your comments, we improved the last paragraph of the Introduction section by defining the circumstances of snakebite so that readers can understand the goals of this study. To let readers know about circumstances, we listed the components under this category (in the parenthesis followed by circumstances). This line appears as "... circumstances (locality of snakebite envenomings, places, and victims' activities when bitten, bitten body parts, time and season of snakebite envenomings, and snakes responsible for envenomings)...".

## Reviewer #5: (No Response)

--------------------

# Editorial and Data Presentation Modifications?

Use this section for editorial suggestions as well as relatively minor modifications of existing data that would enhance clarity. If the only modifications needed are minor and/or editorial, you may wish to recommend “Minor Revision” or “Accept”.

## Reviewer #1:

Accept

**Authors:** Thank you.

## Reviewer #2:

The graph and maps are good and have an important clearance for all the information.

Is it possible to choose a higher resolution? For clarity without pixelation.

**Authors:** Thank you. We have included high resolution-figures now.

## Reviewer #3:

In over all this paper is original and it is higher power of significance for other researchers for further investigation and policy makers to revise their policies. It requires **^#1^**minor revision in the abstract; it must include major variables which were having a significant difference findings.

In the result part it is better to test the **^#2^**normality test of continuous variables and it must be present the data analysis output in terms of averages or means, variance, standard deviation, minimum and maximum values special for age and duration hours.

**^#3^**In the introduction, it is better to include some significant variables.

In methodology it requires to **^#4^**replace missing values with appropriate missing data treatment techniques.

It is necessary to include the **^#5^**method of data collection from traditional snakebite treatment healers. It must clear how to identify species of snakes in traditional snake bite healers.

**^#6^**It is best to remove useless in formation like inadequate transports which is not found in the data analysis outputs.

In the discussion part it must be necessary to **^#7^**discuss for all variables of interest.

It requires minor revision!!

**Authors:** Thank you for your review comments. The responses to your important concerns are given below indicating with #1, #2, ..., etc.

**^#1^**minor revision in the abstract; it must include major variables which were having a significant difference findings:

We improved abstract including major variables which had a significantly different findings.

**^#2^**normality: Our samples represented diverse locations from lowlands to the hills where different venomous snake species distribute and cause snakebite. For example, distribution and density of krait species (nocturnal species) are greater in Terai where they intrude often into residential areas causing snakebite of sleeping people. Next, distribution and diversity of pitvipers is greater in the hills compared to Terai where they cause bite of people active in agrarian areas or forested areas. In this way, our samples represented different locations and different venomous snake species bites. This might have caused non-normality of continuous variables. So, we used nonparametric alternatives for parametric tests.

For the normal distribution of data set, mean and standard deviation/variance would be a target and for the non-normal data set, median and inter-quartile range would give the ideas on nature of data distribution. We have also analyzed interquartile range (IQR) which is more useful for describing median based variables (herein, age, hours, altitudes).

Generally, for the normal distributions, we use to use the standard deviation to determine the percentage of observations that fall at specific distances from the mean. However, that doesn’t work for our non-normal (skewed) distributions. Therefore, the IQR is an excellent alternative to standard deviation. Further, we used IQR to detect any outliers in our data set. Additionally, like the IQR, the median is superb for skewed distributions. Therefore, considering nature of our data distributions of continuous variables, we used median instead of mean and IQR instead of standard deviation.

Further, we calculated 95% confidence interval using the non-parametric statistical analysis using median (instead of mean) to know the range within which expected population median would be placed.

**^#3^**In the introduction, it is better to include some significant variables: Accordingly, we improved the last paragraph of Introduction section.

**^#4^**replace missing values with appropriate missing data treatment techniques: About the missing data values, we mentioned above, too. In our dataset, missing of values were completely at random and unrelated to any of the variables involved in our analysis. Therefore, we adopted a complete case analysis [1]. This was mentioned in the 7th paragraph of the Data analysis subsection of Method section.

**^#5^**method of data collection from traditional snakebite treatment healers: We did not collect data from traditional snakebite treatment healers. In the method section, we mentioned that the news was also collected while determining the localities of traditional snakebite treatment healers for a study which was conducted under my leadership. We think that we stated about data collection strategies clearly.

**^#6^**It is best to remove useless in formation like inadequate transports which is not found in the data analysis outputs:

For the clarity to the readers, we removed "inadequate access to transport when" from the first paragraph of the discussion. Previously, we used the term "inadequate access to transport when" to show that snakebite at night would be an obstacle because there would be difficulties to access proper transport.

**^#7^**discuss for all variables of interest: We greatly improved the discussion part including all variables of interest.

## Reviewer #4:

The study needs minor revisions to address the issues in the general comment and methodology section.

**Authors:** Thank you. We improved Methods section following your comments as well. Also, we considered your other general comments and improved the manuscripts.

## Reviewer #5:

Accept

**Authors:** Thank you Reviewer#5.

--------------------

# Summary and General Comments

Use this section to provide overall comments, discuss strengths/weaknesses of the study, novelty, significance, general execution and scholarship. You may also include additional comments for the author, including concerns about dual publication, research ethics, or publication ethics. If requesting major revision, please articulate the new experiments that are needed.

## Reviewer #1:

The manuscript presents an innovative study that casts light on a critical public health issue in Nepal involving the incidence of venomous snake bites, which result in an alarming number of annual deaths. Nonetheless, this study emphasizes the significance of epidemiological mapping as an effective strategy for reducing fatalities and protecting at-risk populations, particularly children.

**Authors:** Thank you Reviewer#1 for your critical review comments.

## Reviewer #2:

Major and minor points and Additional tables must be added.

Information has been provided in the "Attachments" Section.

Last Comment:

-The published media news is influenced by lies, exaggeration and rumors. How do you verify that? Please provide more details, and Explain that.

**Authors:** Thank you. Following your advice and template of table, we developed two new tables (Table 3 and Table 4). Also, we improved Table 2 greatly.

In my experience in Nepal, I found that media news related to political issues are sometimes exaggerated and rumoured, too. But, snakebite envenomings, deaths and morbidity are humanitarian issues. Nepal Government provides antivenom to the Nepali citizen free of costs. Medical personnel do not expect any benefits from victims of snakebites in Nepalese communities. So, we did not think that news related to snakebite issues is influenced by lies, exaggeration and rumors.

## Reviewer #3:

The strength of the study, is it is noble, highly significant, and the methodology of data collecting strategies, I mean how to data to be included and excluded for the analysis is very interesting and appreciated. Ii also very good in abstract format even if it **^#1^**requires minimum revision the way it has structured is get high credit. But it requires to include more significant outputs from the data analysis either some discussion or conclusion part. in the output part is is so attractive and clear for any one who reads this paper.

But is important to test the **^#2^**normality of the continuous variables in the data analysis since in the methodology part it talks about that continuous variables are not normally distributed but not exist in the result part.

All the **^#3^**occupation of snakebite variable must be describe in the result section. other wise it is so attractive in clear and sufficient to test the objectives of the study. in the discussion part it is so great the way that the result has been discussed with other authors. it is so good more over to **^#4^**discuss for all study variables of interest like the length of times between snakebite and declaration of deaths.

In conclusion part is important for based on all **^#5^**significant variables. otherwise it is so nice. finally you have cited for all references and I want to appreciate for this even if few references are outdated.

**Authors:** Thank you.

**^#1^**requires minimum revision: We improved discussion, conclusions and abstract following review comments.

**^#2^**normality of the continuous variables in the data analysis since in the methodology part it talks about that continuous variables are not normally distributed but not exist in the result part: We discussed about the normality above. To present these non-normally distributed data in continuous variables (included in this study) in result section, we developed a new figure showing box plots and histogram superimposed by density curve for altitudes and hours.

**^#3^**occupation of snakebite variable must be describe in the result section: We mentioned it in result and discussion section in support of Table 2, 3, and 4..

**^#4^**discuss for all study variables of interest like the length of times between snakebite and declaration of deaths: We discussed it in the last paragraph of the discussion section.

**^#5^**significant variables: We also improved conclusion part based on all significant variables.

## Reviewer #4:

The study mentioned at the beginning of the article was conducted in a specific region of Nepal called Terai, but the **^#1^**title needs to reflect this information (110-113).

Several statements are difficult to understand throughout the article and contain **^#2^**grammatical errors. The sentence structures in these statements need to be reviewed. Grammatical errors and hard-to-read sentences can be found in various statement numbers, including 23-24, 30-32, 50-51, 71-74, 112-113, 367-370, 387-391, 398-399, 437-438, and 527-529.

**^#3^**Conducting a thorough review of the article's grammar would be appropriate.

The word **^#4^**"useless" in statement 42 is offensive, and a more appropriate term should be used.

**^#5^**Statement 91-92 is insignificant unless the author wants to highlight the importance of obtaining data from these media outlets.

The authors want to provide the biological classification and common names of the snakes under study. However, their use should be clearly stated to avoid confusion. There should be **^#6^**consistency in the use of common names or biological classification for easy understanding, particularly for non-experts in the field of study. These inconsistencies can be found in statements 39, 55, 133-135 and some parts of the article.

Using **^#7^**synonyms for "envenom" and "novel" would make the article more enjoyable, as these words are repeated frequently. Although they may be keywords used in the search strategy, consider using other words with similar meanings to interest readers.

**^#7^**Some statements are out of place and should be mentioned under the inclusion and exclusion criteria (197, 208-209).

**Authors:** Thank you for the general review comments. We have responded below following superscript of #1, #2, etc.

**^#1^**title needs to reflect this information: While stating the problems of epidemiological study of snakebites, we raised the methodological incompleteness of a recent cross-sectional survey of snakebites carried between 30th Nov 2018 and 7th May 2019 in the 23 districts of Terai region of Nepal [6] in the first paragraph of Introduction section. It is because the authors excluded towns and cities of Terai where snakebite envenomings and associated mortalities are frequently reported [10-12]. This does not mean that we focused to analyse data from these 23 districts from Terai region of Nepal. Our dataset covered snakebite cases from Terai, hills, and mountains (Figure 1 and 5). So, we did not compare and correlate our data with the findings from that study [6]. So, in our understanding the title need to reflect entire Nepal not Terai region of Nepal as "...News Media-Reported Snakebite Envenoming in Nepal ...".

**^#2^**grammatical errors: We corrected these errors as:

23-24, We removed "To our knowledge" because we are confident that nobody has	analyzed media reported snakebites in Nepal yet.

30-32, we replaced gender by sex.

50-51, we replaced situation with situations ".....to understand the epidemiological	situations in Nepal like poor resource country"

71-74, We replaced Nov with November.

112-113, We replaced envenoming with envenomings and "the altitudinal gradients"	with "the altitudes". Now this line appears as " to determine the distribution of	snakebite envenomings along the altitudes and the climatic zones "

367-370, We replaced ] with small bracket in "Nepal [10]]" and replaced " cultures"	with " culture".

387-391, we replaced "high activity" with "intensive activities...".

398-399, Now, this sentence appears as "Therefore, our findings suggested need of	designing a large-scale, nationally representative epidemiological survey.".

437-438, Now, this sentence appears as "Overall, the snake bitten upper and lower	extremities suggest that extremities disturbed snakes which in turn caused	snakebites.".

and 527-529. We improved this section, too. It appears as " This improvement in	treatment-seeking behavior in Nepal signifies the need of analyzing	community- and hospital-based snakebites altogether [3] to understand the	whole	gamut of burden due to snakebite envenomings and associated	consequences.".

**^#3^**Conducting a thorough review of the article's grammar would be appropriate: Previous version of this article was thoroughly reviewed by Prof. Elda Sanches to improve the usage of grammar in this article. We addressed the grammar issues raised by all reviewers. Further, we thoroughly pored over to improve the grammar and syntax used in this manuscript.

**^#4^**"useless" in statement 42 is offensive: We replaced "useless or harmful" with "non-recommended" in the abstract.

**^#5^**Statement 91-92: We removed these lines.

**^#6^**consistency in the use of common names or biological classification for easy understanding, particularly for non-experts in the field of study: We realized it and mentioned common names before the biological names to make non-experts in the field of study easy to read and understand about the names of snake involved in envenomation in Nepal.

**^#7^**synonyms for "envenom" and "novel" would make the article more enjoyable:

We used new and pristine for the "novel" in additional two instances. We used synonym "envenomated" for "envenomed" in some cases. Envenoming is the major key word in this study and we did not get its synonyms for this term. This manuscript follow up American English style and envenomings is often use in this style. Envenomation is used often in British English. So, we did not mix these synonyms.

**^#7^**Some statements are out of place: We deleted these statements from the improper sections and carried to the inclusion and exclusion criteria.

## Reviewer #5:

Participant with insufficient data could have been excluded from the study (eg. those without age, sex and other vital information)

Moreover taking data from newspapers, television and radio could cause duplicates because one info can be channeled through all the three medium.

**Authors:** Thank you for your comments. We defined "Inclusion and exclusion criteria" in Method section and attempted to include case reports with sufficient data such as demographics, circumstances of the bite, prehospital care, and outcome, geographic locations, etc. To understand the situation of snakebite envenomings more precisely, we included only sufficiently defined case reports. If we removed cases without age and sex, other vital information would be removed. Rather we removed all missing values while analysing data.

We excluded those news reporting only incident data, STCs or antivenom shortage, venoms, antivenom production, or expert opinions or recommendations on snakes and/or snakebites, the "Naagpanchami" (a snake festival celebrated by Hindus), familiarizing snake rescuers, involvement of traditional snakebite healers (TSHs) in snakebite care but without case reports. In order to make readers clearer, we excluded this information although they are vital to understand the snakebite epidemiology.

Your expectation of possible duplication was existed in our study. There was several duplicated news which we declined if they reported verbatim news. We have mentioned about the duplicated news in the second paragraph of "Data collection and management" of Methods section in this manuscript greater in detail.

--------------------

# PLOS authors have the option to publish the peer review history of their article (what does this mean?).

If published, this will include your full peer review and any attached files.

If you choose “no”, your identity will remain anonymous but your review may still be made public.

Do you want your identity to be public for this peer review?

For information about this choice, including consent withdrawal, please see our Privacy Policy.

**Reviewer #1:** Yes: Raul Afonso Pommer-Barbosa

**Reviewer #2:** Yes: Imad Addin Almasri

**Reviewer #3:** Yes: Asmare Adane Mekonen

Reviewer #4: No

**Reviewer #5**: Yes: Manfred Dakorah Asiedu

**Authors:** Thank you all reviewers for your constructive comments. Following your advice and comments, we greatly improved this manuscript.

# Figure Files:

While revising your submission, please upload your figure files to the Preflight Analysis and Conversion Engine (PACE) digital diagnostic tool, https://pacev2.apexcovantage.com. PACE helps ensure that figures meet PLOS requirements. To use PACE, you must first register as a user. Then, login and navigate to the UPLOAD tab, where you will find detailed instructions on how to use the tool. If you encounter any issues or have any questions when using PACE, please email us at figures@plos.org.

**Authors:** We have uploaded five PLOS_PACE Corrected figures. We hope that these figures have sufficient quality for the PLOS NTD journal.

# Data Requirements:

Please note that, as a condition of publication, PLOS' data policy requires that you make available all data used to draw the conclusions outlined in your manuscript. Data must be deposited in an appropriate repository, included within the body of the manuscript, or uploaded as supporting information. This includes all numerical values that were used to generate graphs, histograms etc.. For an example see here: http://www.plosbiology.org/article/info%3Adoi%2F10.1371%2Fjournal.pbio.1001908#s5.

**Authors:** We used three supplementary tables which are uploaded in the separate file name. Next, we uploaded tab delimited text file which contains all numerical values that were used to generate graphs, histograms, etc, using R statistical program [the file name is " 1_296_107_MediaReported_Snb_inclTSHs_230728_R_.txt"]. We do not have own web page. So, please, make this dataset available to the reviewers and readers of this article.

# Reproducibility:

To enhance the reproducibility of your results, we recommend that you deposit your laboratory protocols in protocols.io, where a protocol can be assigned its own identifier (DOI) such that it can be cited independently in the future. Additionally, PLOS ONE offers an option to publish peer-reviewed clinical study protocols. Read more information on sharing protocols at https://plos.org/protocols?utm_medium=editorial-email&utm_source=authorletters&utm_campaign=protocols

__________________________________________________

In compliance with data protection regulations, you may request that we remove your personal registration details at any time. (Use the following URL: https://www.editorialmanager.com/pntd/login.asp?a=r). Please contact the publication office if you have any questions.

**Authors:** We do not have study protocols. Our well-described methods should have reproducibility of this research article.

References

1. Heymans MW, Twisk JW (2022) Handling missing data in clinical research. Journal of Clinical Epidemiology 151: 185-188.

2. Pandey DP (2007) Epidemiology of snakebites based on field survey in Chitwan and Nawalparasi Districts, Nepal. Journal of Medical Toxicology 3: 164–168.

3. Armstrong LJ, Cynthia S, George M, Zachariah A (2019) Comparing community and hospital data of snakebite in North Bihar: a community incidence survey and a parallel hospital-based clinical study. Tropical Doctor 49: 285-292.
